# High-density linkage map construction and identification of loci regulating fruit quality traits in blueberry

**DOI:** 10.1038/s41438-021-00605-z

**Published:** 2021-08-01

**Authors:** Molla F. Mengist, Hamed Bostan, Elisheba Young, Kristine L. Kay, Nicholas Gillitt, James Ballington, Colin D. Kay, Mario G. Ferruzzi, Hamid Ashrafi, Mary Ann Lila, Massimo Iorizzo

**Affiliations:** 1 Plants for Human Health Institute, North Carolina State University, Kannapolis, NC USA; 2 Department of Horticultural Science, North Carolina State University, Raleigh, NC USA; 3 David H. Murdock Research Institute, Kannapolis, NC USA; 4 Department of Food Bioprocessing and Nutrition Sciences, North Carolina State University, Raleigh, NC USA

**Keywords:** Plant breeding, Plant genetics

## Abstract

Fruit quality traits play a significant role in consumer preferences and consumption in blueberry (*Vaccinium corymbosum* L). The objectives of this study were to construct a high-density linkage map and to identify the underlying genetic basis of fruit quality traits in blueberry. A total of 287 F_1_ individuals derived from a cross between two southern highbush blueberry cultivars, ‘Reveille’ and ‘Arlen’, were phenotyped over three years (2016–2018) for fruit quality-related traits, including titratable acidity, pH, total soluble solids, and fruit weight. A high-density linkage map was constructed using 17k single nucleotide polymorphisms markers. The linkage map spanned a total of 1397 cM with an average inter-loci distance of 0.08 cM. The quantitative trait loci interval mapping based on the hidden Markov model identified 18 loci for fruit quality traits, including seven loci for fruit weight, three loci for titratable acidity, five loci for pH, and three loci for total soluble solids. Ten of these loci were detected in more than one year. These loci explained phenotypic variance ranging from 7 to 28% for titratable acidity and total soluble solid, and 8–13% for pH. However, the loci identified for fruit weight did not explain more than 10% of the phenotypic variance. We also reported the association between fruit quality traits and metabolites detected by Proton nuclear magnetic resonance analysis directly responsible for these fruit quality traits. Organic acids, citric acid, and quinic acid were significantly (*P* < 0.05) and positively correlated with titratable acidity. Sugar molecules showed a strong and positive correlation with total soluble solids. Overall, the study dissected the genetic basis of fruit quality traits and established an association between these fruit quality traits and metabolites.

## Introduction

Blueberries are well recognized as a rich source of health-promoting phytochemicals, which have in part contributed to a rapid increase in consumer demand and production over the past 15 years^1–4^. In the USA, per-capita blueberry consumption increased from 0.18 kg/person in 2002 to 0.57 kg/person in 2011 with a 21.66% annual growth rate for this specific period^5^. Polyphenols or bioactives such as flavonoids (anthocyanins, flavanols, and flavonols) and non-flavonoids such as phenolic acids are found in large amount in blueberry. Clinical evidence suggests that sufficient intake of blueberries provides multiple health benefits including lowering blood pressure, protecting against heart attack, preventing cancer, improving mental health and managing diabetes^1,2,6^. These health benefits associated with blueberry consumption contributed to the increased consumption. In recent years, the blueberry industry recognized that after a decade of significant production expansion and growth, the blueberry market in North America has matured, whereby both the industry and consumers are becoming more selective and quality-driven^7,8^. Indeed, multiple studies in blueberry reported that fruit quality (FQ) traits, including size (FW), total soluble solid content (TSS), titratable acidity (TA), and texture influence consumer preferences^1–4,9–13^. In particular, consumers cited flavor and sweetness as positive characteristics, and TSS and TA as the most and least important characteristics associated with overall consumer preferences for blueberries, respectively^4,14^. In fact, the balance between sugars and acids is determinant factor for berry taste. Sweet berries do not necessarily have high sugar content, instead they may retain lower level of acids, which results in the higher the sugar/organic acid ratio^4,13,14^.

While some cultivars meet consumer preferences, a wide variation for FQ traits exists among blueberry cultivars. As a result, blueberries available on the market represent a blend of multiple cultivars, and the variation for FQ traits makes the consumer experience inconsistent across multiple purchases. In turn, this can affect consumer re-purchasing frequency and overall consumption^4,7,8,14^. Developing strategies that can increase the blueberry production that meet consumer preferences will be critical for sustaining the consumption growth.

The development of new cultivars has played and will continue to play a major role in the growth of the blueberry market in North America and worldwide. In the last two decades, breeding programs have developed improved cultivars that have supported the expansion of blueberry production into new growing areas, such as low-chill regions and the southern hemisphere. Today the industry and consumers are quality driven, and genetic gains for fruit quality traits will be a key factor to sustain the growth of the blueberry industry^7,8^.

Blueberry breeding activities strongly rely on phenotypic-based selection for multiple traits following multiple rounds of crossing and testing in multiple environments. The use of wild species in blueberry breeding programs is common and has contributed to blueberry improvement with respect to a wide range of important traits including resistance to biotic and abiotic stress, fruit quality and adaptation to new environments^15–18^. However, the process of breeding a new variety solely based on phenotypic selection takes a long time (10–20 years), is expensive and somewhat inefficient^17,19^. As a result, breeders are only able to select for a limited number of traits. Modern plant breeding approaches such as genomic-assisted breeding must be incorporated into traditional breeding programs to meet the current consumer’s preferences and accelerate breeding activities, especially for FQ traits.

The use of advanced molecular tools to facilitate breeding for economically important traits including FQ rely on the characterization of the existing blueberry germplasm^1–3,11^ and the study of the genetic mechanisms controlling these traits. Multiple studies demonstrated significant phenotypic variability for FQ traits within blueberry germplasm, and the traits have also shown moderate to high genetic heritability^1–3,11,20,21^.

Quantitative trait loci (QTL) mapping has been an important method to study the genetic mechanisms and identify genomic regions and genes that control traits of interest including FQ traits^21,22^. Despite the importance of FQ traits, only few studies have reported QTLs for FQ traits in blueberries^11,21^. This is partly due to limited genomic resources and tools that are important for the investigation of the genetic basis of important FQ traits in blueberry^11^.

Recent advances in next generation sequencing (NGS), high-throughput genotyping platforms, reference genomes, and statistical tools offer the development of a sufficient numbers of molecular markers, high-density genetic maps, and increased power for QTL detection in tetraploid species including blueberry^21–25^. Hence, the objectives of this study were to develop a high-density linkage map and to investigate the genetic control of FQ traits including FW, TSS, TA, and pH through QTL analysis in an F_1_ mapping population. The study also investigated the association between FQ traits and organic acids, sugars, and amino acids.

## Results

### Phenotypic data

**Fig. 1:**
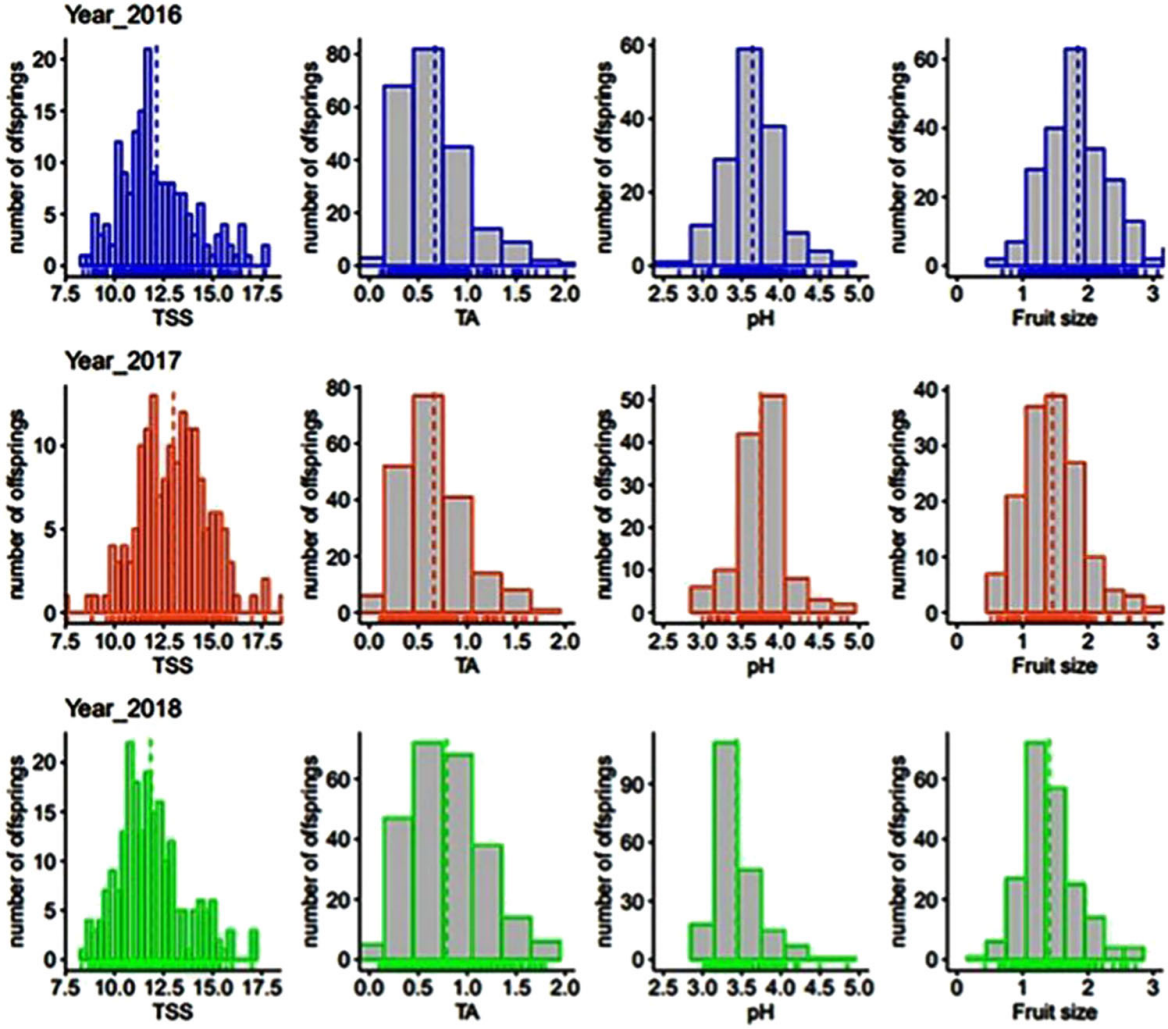
Phenotypic distribution of FQ traits over three years. TSS, total soluble solids; TA titratable acidity

Extensive phenotypic variation was observed for all FQ traits for three consecutive years (2016–2018). Similar levels of trait variability were observed for three years, with an approximate 1.7, 2, 6 and 10-fold phenotypic variation for pH, TSS, FW and TA, respectively (Supplementary Table 1). Analysis of variance (ANOVA) showed a significant effect of genotype, year and genotype by year interaction for all the FQ traits in this study (Supplementary Table 2). Trait distributions were somewhat similar for all three years. The FQ traits, TSS, FW and pH had a near-normal distribution, suggesting a polygenic genetic control. However, the distribution of TA in the population was skewed towards the lower acidity values (Fig. 1). Broad sense heritability (H^2^) estimates were 46% for TSS, 52% for pH, 60% for TA and 74% for FW (Supplementary Table 1). TA had the highest phenotypic variability while FW showed a higher broad sense heritability among the traits considered in this study.

**Fig. 2:**
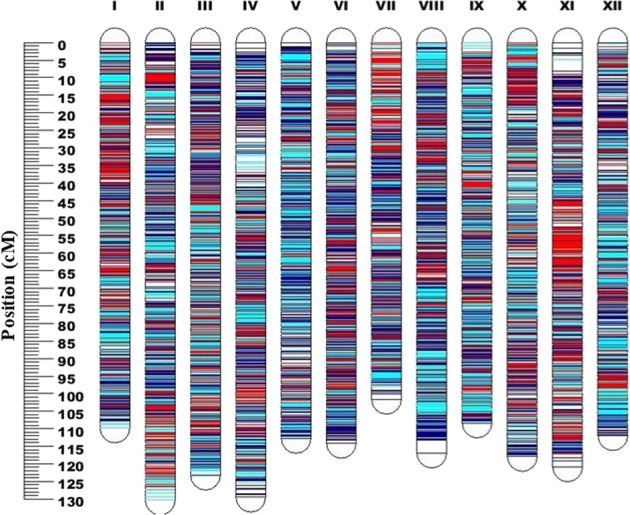
Distribution of SNP markers along the 12 linkage groups (LG) (I–XII) mapped in the RxA mapping population. SNP positions are marked in red from ‘Arlen’ (**A**), blue from ‘Reveille’ (R) and cyan (common markers). LG numbers (I–XII) were assigned based on the corresponding chromosome numbers (1–12) on the published tetraploid blueberry genome^25^

Correlation analysis revealed that TA and pH were strongly and negatively correlated across three years (Supplementary Fig. 1). However, the correlations between traits did not show a similar pattern over the years. For 2016 data, TSS was negatively correlated with pH. Whereas FW was significantly and negatively correlated with TSS and pH for 2017 data (Fig. 2). Overall, the correlation analysis revealed a clear pattern for TA and pH. The relationship between other traits were environmentally dependent (Supplementary Fig. 1).

### High density linkage map construction

A total of 17,438 single nucleotide polymorphisms (SNP) markers were used for linkage map construction. The SNP markers were composed of 8,232 simplex (AAAB x AAAA), 2,655 duplex (AABB x AAAA) and 6,551 common markers (AAAB x AAAB; AABB x AAAB). On average, 47% of the markers were simplex. Across the chromosomes, the composition of simplex markers to the total markers in each linkage map varied from 43% on chromosome 12 to 50% on chromosome 10 (Table 1).

**Table 1:** Distribution of SNPs into genotype classes and summary of integrated map of Reveille x Arlen mapping population

Chr.	No. of mapped SNP	SxN^1^ (#)	DxN^1^ (#)	SxN^2^ (#)	DxN^2^ (#)	Common Markers (#)	% of simplex SNPs	Map length (cM)	Average inter-loci Distance (cM)
1	1494	283	69	440	167	535	48.39	109.8	0.0730
2	1300	329	90	263	101	517	45.54	130.31	0.1000
3	1644	382	73	369	169	651	45.68	123.23	0.0750
4	1339	350	118	292	106	473	47.95	129.48	0.0968
5	1393	379	60	301	58	595	48.82	112.73	0.0810
6	1749	448	135	389	193	584	47.86	114.17	0.0650
7	1267	281	130	334	94	428	48.54	101.61	0.0803
8	1798	477	142	382	110	687	47.78	116.92	0.0650
9	1426	280	81	361	108	596	44.95	108.36	0.0760
10	1346	307	79	371	111	478	50.37	117.89	0.0880
11	1254	241	108	357	192	356	47.69	120.85	0.0964
12	1428	333	96	283	65	651	43.14	111.97	0.0785
Total	17438	4090	1181	4142	1474	6551	47.21	1397.32	0.0812

^1^indicate simplex (SxN) or duplex (DxN) markers from Arlen; ^2^indicates simplex (SxN) or duplex (DxN) markers from Reveille. Common SNPs represent simplex by simplex (SxS), duplex by simplex (DxS), simplex by duplex (SxD), duplex-by-duplex (DxD). Chr., chromosome

The final map covered a total of 1,397 cM, with an average inter-locus distance of 0.0182 cM. The average length of a linkage group was 116 cM, ranging from 101.6 cM on chromosome 7 to 130.3 cM on chromosome 2 (Table 1, Fig. 2). The MDS diagnostic plot indicated that the map data did not have large gaps and outliers (Supplementary Fig. 2). Furthermore, the SNP markers were uniformly distributed across eight homologous chromosomes of each linkage group except for ‘Arlen’ chromosome 1. On chromosome 1, half of two homologous representing the parent ‘Arlen’ were not covered by any marker (Supplementary Fig. 3). Overall, the marker coverage/density on the integrated map (Table 1, Fig. 2) was uniform. In addition, it was possible to identify all the 96 homologous chromosomes (12 chromosome × two parents × four copies of homologs) that are expected from the tetrasomic species of blueberry (Supplementary Fig. 3).

Alignment of the integrated map (cM) with the physical map (Mb) of tetraploid reference genomes revealed that the linkage map was highly collinear with the tetraploid reference genome (Supplementary Fig. 4). Minor rearrangements were identified while examining the collinearity between the genetic linkage map and tetraploid genome in regions of chromosomes 5, 6, 7, and 10 with low recombination, likely centromeric, and some degree on the telomeric regions of chromosomes 2, 3, 5, 7 and 9 (Supplementary Fig. 4).

### QTL mapping for fruit quality traits

**Fig. 3:**
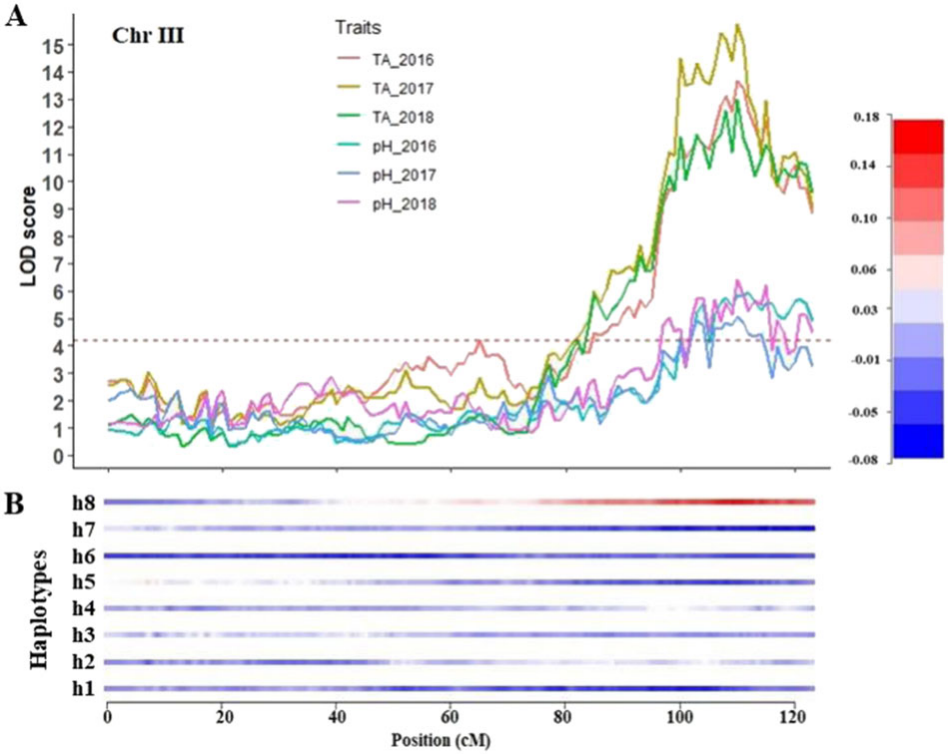
QTLs identified for titratable acidity and pH on linkage group 3 and the effect of haplotypes relative to the overall phenotypic mean. **A** Representation of six QTLs for titratable acidity and pH in linkage group 3 (representing chromosome 3) detected over three years; **B** heatmap legend shows the effect of each homolog relative the overall phenotypic mean performance. The h1-h8 represents the eight homologs with the first four homologs (h1-h4) from the parent ‘Arlen’ and the other four homologs (h5-h8) from the parent ‘Reveille’. Chr, chromosome

QTLs controlling TA in blueberry were identified on chromosome 3, 4, and 5. A major-effect QTL was detected on chromosome 3 with LOD scores of 14, 16 and 13 for three consecutive years, 2016–2018, respectively. This QTL explained 22%, 28%, and 19% of the phenotypic variance (PV) for 2016–2018, respectively and was associated with increasing acidity values. Analysis of the QTL genotype means using the simple model indicated that a simplex allele on homologous 8 (h8) of ‘Reveille’ had the lowest Schwarz Information Criteria (SIC) compared to the SIC of the full (additive) model (Table 2, Fig. 3). The locus is located around 110 cM, which corresponds to position 37.9 Mb of the tetraploid blueberry genome, chromosome 3 (VaccDscaff 9). A second QTL identified on chromosome 5 was detected for three years, 2016–2018 with LOD scores of 6.6, 8.2, and 5.2, respectively. The QTL explained 10%, 14 and 7% of the PV for 2016, 2017, and 2018, respectively. An additional minor effect QTL on chromosome 4 was detected for the 2016 cropping season (Table 2).

**Table 2:** Summary of QTLs identified for fruit quality traits in the Reveille x Arlen mapping population

Trait	Chr	LOD	R^2^	Position (cM)	Best simple model	Scaffold (S) and position (Mb)	Effect
FW_2017	2	4.43	8.83	64	S15	(S25, 27.4)	-ve
FW_2018	2	6.27	10.14	56	S15	(S25, 27.4)	-ve
TSS_2017	2	4.56	8.60	33	H1	(S68, 0.75)	-ve
FW_2018	3	4.99	8.00	90	V57	(S37, 25.71)	-ve
FW_BLUE	3	5.48	6.00	82	H5	(S4, 32.92)	-ve
pH_2016	3	5.93	13.00	112	H8	(S9, 37.9)	-ve
pH_2017	3	5.06	13.00	110	H8	(S9, 37.9)	-ve
pH_2018	3	6.40	11.00	110	H8	(S9, 37.9)	-ve
TA_2016	3	13.67	22.00	110	H8	(S9, 37.9)	+ ve
TA_2017	3	15.74	28.00	110	H8	(S9, 37.9)	+ ve
TA_2018	3	13.00	19.00	110	H8	(S9, 37.9)	+ ve
TSS.TA_2016	3	7.15	16.29	115	H8	(S9, 37.9)	-ve
TSS.TA_2017	3	7.57	19.00	110	H8	(S9, 37.9)	-ve
TSS.TA_2018	3	8.29	14.00	110	H8	(S9, 37.9)	-ve
FW_2016	4	4.67	7.00	105	S35	(8.0, 39)	-ve
FW_2017	4	4.18	8.00	76	H2	(S38, 18.37)	-ve
FW_2018	4	5.44	9.00	77	V13	(S38, 18.50)	-ve
pH_2016	4	5.03	10.28	114	S16	(S38, 5.93)	+ ve
pH_2017	4	4.37	10.00	101	S26	(S37, 22.3)	+ ve
TA_2016	4	4.47	6.10	98	S27	(S37, 26.67)	+ ve
pH_2016	5	4.44	8.70	12	H3	(S7, 2.72)	+ ve
pH_2018	5	4.88	8.00	13	S38	(S7, 2.72)	+ ve
TA_2016	5	6.64	10.25	6	H3	(S7, 2.72)	-ve
TA_2017	5	8.21	14.00	9	S38	(S7, 2.72)	-ve
TA_2018	5	5.18	7.00	13	S38	(S7, 2.72)	-ve
FW_2016	7	4.58	6.50	9	H8	(S12, 3.1)	+ ve
pH_2017	7	5.07	13.00	34	T15	(S41, 1)	+ ve
TSS_2016	7	7.52	15.00	26	D58	(S23, 30.3)	-ve
TSS_2017	7	13.34	28.60	26	H5	(S23, 30.3)	-ve
TSS_2018	7	8.20	14.00	24	H5	(S23, 30.3)	-ve
FW_2018	8	5.30	8.20	45	V58	(S32, 27.72)	+ ve
pH_2018	8	4.83	8.00	49	D34	(S30, 24.58)	-ve
pH_BLUE	8	6.25	8.39	75	H2	(S32, 15.65)	+ ve
FW_2016	10	4.75	7.00	57	S15	(S44, 18.19)	+ ve
TSS_2016	10	7.60	16.00	79	S46	(S44, 9.4)	+ ve
TSS_2017	10	6.88	14.00	69	S46	(S44, 9.4)	+ ve
TSS_2018	10	4.86	7.00	54	S46	(S44, 9.4)	+ ve
FW_2016	12	4.73	7.00	90	H2	(S43, 7.9)	-ve
FW_2017	12	4.74	9.80	90	S36	(S43, 7.9)	-ve
FW_BLUE	12	6.74	8.20	90	H2	(S43, 7.9)	+ ve

Chr., chromosome number; LOD, threshold values of logarithms of odds (LOD) for the QTL calculated by TetraploidSNPMap; R^2^, phenotypic variance explained by the QTL; BLUE, best linear unbiased estimate; H1, simplex marker on homolog 1; S38, double simplex marker on homologs 3 and 8; V13, duplex as codominant variate on homologs 1 and 3; T15, dominant double simplex; D58, dominant duplex markers on homologs 5 and 8; TSS.TA, ratio of TSS to TA. Simplex models are indicated as homologs h1-h4 from ‘Arlen’ and h5-h8 from ‘Reveille’. Effect represents the direction of the QTL effect on the trait performance (increasing = +ve and reducing -ve). Scaffold (S) and physical position (Mb) of the markers spanning the QTL peak refer to the Draper genome^25^

A total of five QTLs were identified for pH, one on each chromosome 3, 4, 5, 7 and 8. A QTL identified on chromosome 3 was stable across three years, with LOD scores of 5.9, 5 and 6.4, and explained 13%, 13 and 11% of the PV for 2016–2018, respectively (Table 2). A second QTL was found on chromosome 4 for two years (2016 and 2017) and explained 10% of the PV for both years. Another QTL on chromosome 5 was detected for two years (2016 and 2018) and explained 8% of the PV (Table 2). In addition to this, the two QTLs on chromosomes 7 and 8 were detected for one year, year-2017 and year-2018, respectively. Consistent with the correlation analysis, three out of the five QTLs detected for pH co-localized with that of TA (Table 2), while the other two QTLs on chromosomes 7 and 8 were specific for pH (Table 2).

**Fig. 4:**
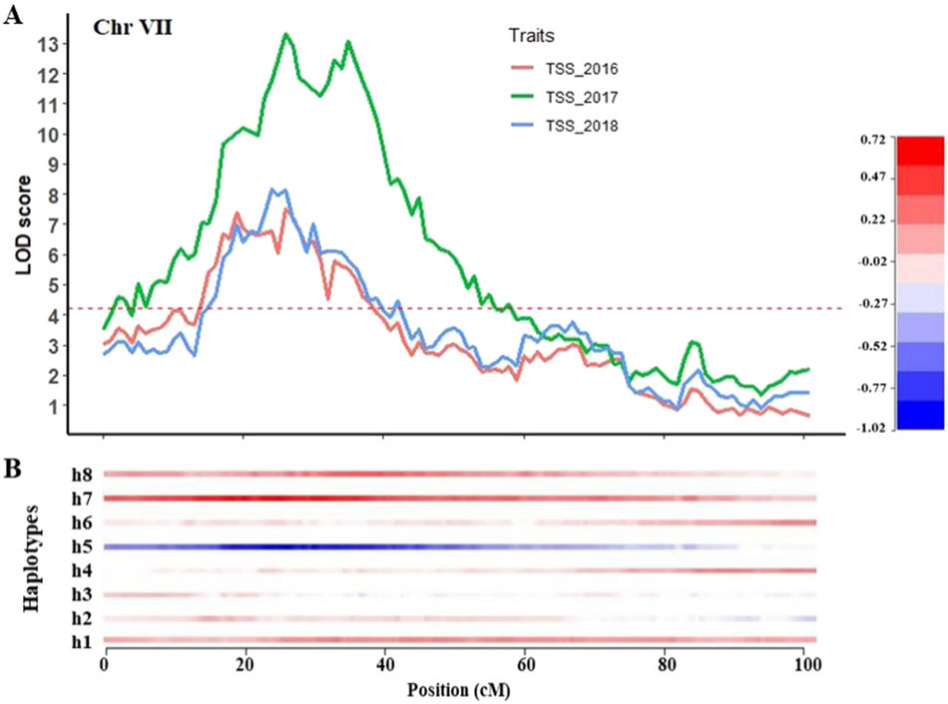
QTLs identified for total soluble solids on linkage group 7 and the effect of haplotypes relative to the overall phenotypic mean. **A** Representation of three QTLs for total soluble solids detected in linkage group 7 (representing chromosome 7) over three years; **B** heatmap legend shows the effect of each homolog relative the overall phenotypic mean performance. The h1-h8 represents the eight homologs with the first four homologs (h1-h4) from the parent ‘Arlen’ and the other four homologs (h5-h8) from the parent ‘Reveille’. Chr, chromosome

For TSS, three QTLs were detected one on each chromosome 2, 7 and 10. The QTL on chromosome 7 was stable over three years, with LOD scores of 7.5, 13.3 and 8.2, and explained 15%, 28 and 14% of PV for years 2016–2018, respectively (Table 2, Fig. 4). Analysis of the QTL genotype means using the simple model showed that a simplex allele on homologous 5 (h5) of ‘Reveille’ had the lowest SIC compared to the SIC of the full (additive) model and was associated with reducing the TSS values (Table 2). This QTL is located around 26 cM of the linkage map, which is 30.3 Mb of the Draper genome of VaccDscaff23 (Fig. 4; Supplementary Table 3). Similarly, the QTL on chromosome 10 was stable across three years and explained 16%, 14 and 7% for years 2016–2018, respectively. Analysis of the QTL genotype means using the simple model showed that a double simplex allele on homologs 4 (h4) of ‘Arlen’ and 6 (h6) of ‘Reveille’ had the lowest SIC compared to the SIC of the full (additive) model. This QTL was associated with increasing TSS value (Table 2), and was located at 9.4 Mb of the Draper genome of VaccDscaff44 (Supplementary Table 3). Another QTL on chromosome 2 was detected for one year (Table 2).

For FW, seven QTLs were detected one on each chromosome 2, 3, 4, 7, 8, 10, and 12 (Table 2). A QTL on chromosome 4 was detected for three years and explained 7%, 8%, and 9% of the PV for years 2016–2018, respectively (Table 2). A QTL on chromosome 2 was identified for two years, with LOD scores of 4.43 and 6.33, and explained 9 and 10% of the PV for the years 2017 and 2018, respectively. Similarly, a QTL on chromosome 12 was detected in 2016 and 2017 and explained 7% and 10% of the PV, respectively (Table 2). Four other QTL one on each chromosome 3, 7, 8, and 10 were also detected for one year (Table 2). Overall, three out of the seven QTLs detected for FW were stable over two years. However, none of these QTLs explained more than 10% of the PV. Although we did not observe a significant phenotypic correlation between FW with other FQ traits, most of the QTLs detected for FW were located in the same genomic regions of the other FQ traits evaluated here (Table 2).

### Metabolome analysis

**Fig. 5:**
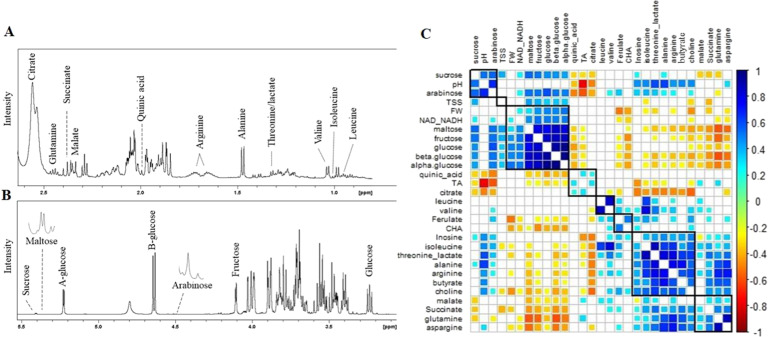
Details of proton nuclear magnetic resonance (^1^H NMR) spectrum of blueberry extract and correlation between fruit quality traits and metabolites. Annotated spectrum shows data acquired for a pooled sample. Selected peaks used for quantitative analysis are labeled in (**A**) upfield region with organic and amino acids, (**B**) midfield region with sugar peaks, (**C**) correlation between FQ traits evaluated in this study, and metabolites detected using ^1^H-NMR across 98 F_1_ Reveille × Arlen genotypes. TA, titratable acidity; TSS, total soluble solids; FW, fruit weight; CHA, chlorogenic acid; NAD-NADH, nicotinamide adenine dinucleotide (NAD) + hydrogen (H)

Metabolic profiling of blueberry extracts revealed over 80 distinct peaks across the Proton nuclear magnetic resonance (^1^H NMR) spectra. A total of 45 annotations were made using reference library data integrated into Chenomx software (Supplementary Table 4). Of these annotations, 24 compounds were annotated with higher confidence (level 2B) based on orthogonal 1D or 2D data from reference databases. A total of 25 metabolites were sufficiently resolved to be quantified (Supplementary Table 4). Among the upfield peaks in the spectra (Fig. 5A), the most intense peaks were citric acid and alanine. The midfield peaks (Fig. 5B) mainly comprised intense and partially overlapping sugar peaks. The sugars that were sufficiently resolved for quantitation were glucose, fructose, sucrose, arabinose and maltose. The most intense peaks in the downfield region (6.0–10 ppm) arose from chlorogenic acid. A singlet peak at 3.19 ppm was annotated as either choline or O-phosphocholine based on an HSQC cross peak (3.21/56.6 ppm) but could not be confirmed further. The doublet at 1.32 ppm was annotated as either lactate or threonine based on an HSQC cross peak (1.33/22.7 ppm) but could not be confirmed further.

### Phenotypic variation of the 25 metabolites

A total of 25 metabolite peaks including seven sugars, five organic acids, and nine amino acids were used for the subsequent analysis. The phenotypic distribution, correlation and PCA analysis were performed based on these metabolites.

Extensive phenotypic variation was revealed for all metabolites in the study. For sugar, there was a 1.5-fold variation between genotypes for all sugars except for sucrose. Sucrose showed a 5.8-fold variation between genotypes (Supplementary Table 5). Amino acids varied between genotypes, ranging from 2.5 to 7.6-fold changes for x-amino butyrate to arginine, respectively. Among organic acids, citric acid followed by ferulate showed the highest phenotypic variation between the genotypes (Supplementary Table 5). The histogram distributions of the metabolites also showed that most of the metabolites had a bimodal distribution (Supplementary Fig. 5), suggesting that few genes may regulate these traits. To compare the composition of metabolites, we first grouped the metabolites into sugars, organic acids and amino acids. We observed that citric acid followed by quinic acid were the most abundant organic acids in blueberry. Similarly, beta glucose followed by fructose were the most abundant sugars whereas, arabinose and maltose were the least abundant sugars in blueberry. Among the annotated amino acids, arginine and 4-amino butyrate were the most abundant whereas, isoleucine and asparagine were the least abundant amino acids observed in blueberry (Supplementary Fig. 6).

### Association between metabolites and fruit quality traits

To further examine the association between metabolites and fruit quality traits, we ran correlation and principal component analysis (PCA) using data from the 2016 cropping season. As expected, citric and quinic acid were strongly and positively correlated with TA, whereas components of sugars were strongly and positively correlated with TSS (Fig. 5C). The correlation analysis also revealed that metabolites were clustered based on their chemical properties (Fig. 5). The PCA analysis also showed that the first two components explained 56% of the variances. The results also showed that citrate is an important variable to discriminate the genotypes and strongly associated with TA. The data also showed that arabinose is an important discriminant variable in the sugar group (Supplementary Fig. 7).

## Discussion

### High-density linkage map construction

High-density genetic maps are a prerequisite for the precise identification, dissection, and quantification of QTLs and map-based gene cloning, and are useful for genomic–assisted breeding^15,21,22,26^. The linkage maps also provide critical information about organization of the genome of the population being evaluated, its recombination landscape and facilitated genome assembly and structural comparative genome analysis within a species^27^. Despite its importance, only two linkage maps are currently available for tetraploid blueberry^21,26^. This is, in part, due to the high cost of genotyping a large number of samples, the few genotyping platforms available for blueberry, and limited tools to estimate dosage and incorporate dosage in linkage map construction. The recent advances in sequencing platforms such as capture-seq, along with the development of new computational tools to estimate SNP dosage and construct linkage maps with high numbers of markers has facilitated closing some of the existing gaps in blueberry genetics. The first linkage map for autotetraploid blueberry was constructed using 99 individuals from an F_1_ mapping population derived from a cross between Jewel and Draper. A total of 1,794 SNP markers and 233 SSR markers were mapped and exhibited segregation patterns consistent with a random chromosomal segregation model for meiosis in an autotetraploid. The linkage map was constructed and included 12 and 20 linkage groups for Draper and Jewel, respectively^26^. The linkage map was fragmented and did not identify all expected 96 homologous chromosomes, 48 for each parent^26^. Most recently, a new linkage map was developed with 237 F_1_ individuals and included 11k SNP markers^21^. This linkage map represented two cultivars named ‘Sweetcrisp’ and ‘Indigocrisp’ and covered all 96 parental chromosomes. The linkage map presented here represents the linkage map with the largest set of markers (17k) and individuals (N = 287) in blueberry^21,26^ and represents the structure of the genome of two additional cultivars (‘Reveille’ and ‘Arlen’) and covered all 96 parental homologous chromosomes.

### QTL mapping for fruit quality traits

The development of blueberry varieties with quality that consistently meets consumer preferences and industry needs is a high priority for the blueberry industry^7^. The balance between sweetness and acidity in blueberry fruits is an important quality criterion of consumer preferences for blueberry fruit and can affect purchasing decisions^4,13,14^. In this article, we described the phenotypic variation of four FQ traits, their genetic basis, and their association with specific metabolites.

Overall consumer preference for blueberry is positively and strongly correlated with sweetness and TSS has been identified as a very good indicator for sweetness and overall consumer acceptance of blueberry fruits^4,13,14^. Strong and positive associations of TSS with sugar molecules such as arabinose, glucose, fructose, and sucrose were established in this study (Fig. 5). We also observed that glucose was the most abundant sugar in blueberries (Supplementary Fig. 5). These results are consistent with the recent findings^13^ that glucose followed by fructose were the most abundant sugar in blueberry cultivars. Despite the moderate phenotypic variation and up to a 2-fold variation for TSS between blueberry accessions was detected in previous studies^1,4^, the genetic basis of TSS has not been fully investigated to date^28^.

In this study, we identified three QTLs that explained from 7 to 28% of variation for TSS, of which two QTLs on chromosomes 7 and 10 were stable across three years. However, the QTL peak position on chromosome 10 was shifted over the years and spanned a wide genomic region (Table 2). The peak position of the TSS QTL on chromosome 7 overlapped over years and was located at 30.3 Mb genomic regions of the Draper genome. Within this region, a UDP-glycosyltransferase (Supplementary Table 3) was identified as a possible candidate gene. UDP-glycosyltransferases are responsible for the metabolic process including the transfer of sugar moieties from activated donor molecules to specific acceptor molecules such as sugars, lipids and nucleic acids^29^. The TSS QTL on chromosome 7 was negatively regulated by the total soluble solids, suggesting that the glucose molecules are transferred to the synthesis of water-insoluble molecules. Indeed, UDP-glycosyltransferases are involved in the synthesis of insoluble solids such as callose and cellulose, so its activity may have resulted in trade off with the other soluble solids^30,31^.

### Blueberry acidity

Titratable acidity is a determinant factor for blueberry sourness^4,13^. According to^4,14^, sourness is negatively associated with overall acceptance by blueberry consumers. In fact, TA and pH are excellent predictors for sourness; therefore, higher acidity is also associated with lower consumer preference^4^. As expected, a strong correlation was observed between TA and pH. This relationship was also reported in our previous study using a diverse set of blueberry accessions^1,2^. However, the two traits (TA/pH) did not show strong and consistent correlation with other traits including FW and TSS over the years. This suggests that the relationship between these traits was strongly environmentally dependent, consistent with the previous reports^1,4^. The presence of significant phenotypic variation for TA/pH in blueberry germplasm was reported in the previous studies^1,4,13^. Furthermore, the moderate broad sense heritability estimated for TA/pH in this study was consistent with previous reports^1^. Given the moderate broad sense heritability and phenotypic variation among blueberry accessions for TA/pH, the genetic basis of the phenotypic variability has not been yet fully exploited in blueberry^1,4,11,13^.

In this study, we reported three QTLs that regulate both TA and pH, and two additional QTLs specific for pH. The QTL for TA/pH located on chromosome 3 was stable across three years and co-localized for both traits. The QTL genotype mean analysis indicated a simplex marker (H8) from the parent ‘Reveille’, was the best fit for the full model. This implies that one haplotype/homologous region inherited from ‘Reveille’ contributes to increased TA. The closest SNP marker associated with this QTL was located at 38 Mb of the Draper genome at scaffold VaccDscaff9 (Supplementary Table 3). Consistent with this result, a recent GWAS study^11^ detected a QTL for pH at the same location at ~ 38 Mb of the Draper genome on chromosome 3, suggesting this QTL is stable across populations. Analysis of the genes predicted in this region allowed us to identify a H ( + )-ATPase that could be a possible candidate gene regulating the pH/TA of blueberry (Supplementary Table 3). Phylogenetic analysis of known H^+^-ATPases confirmed that the blueberry gene (gene 379.23) clustered with the P3A-ATPase subfamily that includes *PH*5-like H^+^-ATPase genes (Supplementary Fig. 8), was responsible for citrate accumulation in plants^32^. In addition to the manual inspection of this region, the protein sequences of five functionally characterized genes, including *PH* from melon^33^, *Ma*, *Ma2*, and *Ma10* from apple^34^, *PpRPH* from peach^9^, regulate pH and TA, and were aligned against the Draper genome. None of these genes aligned to chromosome 3 of the blueberry.

Fruit acidity depends on the composition and levels of organic acids^13,35^. Multivariate analysis in this study revealed a strong association between citrate and pH/TA of blueberry. The results also suggest that citric acid followed by quinic acid are the most abundant organic acids in blueberry. These results are consistent with a recent study on different blueberry cultivars evaluated at two locations in China^13^. A study in a related species, cranberry, showed that TA was strongly and positively correlated with citric and quinic acids. Furthermore, the degree of correlation was higher for TA with citric acid than TA with quinic acid^35^. QTL analysis identified a major-effect QTL for citric acid which has been recently deployed in marker-assisted selection^35,36^. Although we did not investigate the genetic basis of organic acids in this study, our results suggested that citric acid strongly contributes to TA/pH and that the R×A population is segregating for citric and quinic acids. In addition, the candidate gene, *PH*, identified on chromosome 3 is involved in citric acid accumulation. These results open the opportunity to further study the genetic basis of these organic acids and their contribution to TA, pH and to consumer preference.

Fruit weight is an important FQ trait in blueberry breeding. According to ref. ^28^, consumers prefer large-sized berry fruits. Interestingly, this study highlighted that larger-sized berries have higher sugar concentrations than smaller-sized berries (Supplementary Fig. 7). In addition, our previous study indicated that TSS was moderately correlated with fruit size. These results suggest that selection for larger-sized berries could facilitate selection for high sugar content and TSS, which are associated with sweetness and overall consumer liking. On the other hand, FW was negatively correlated with multiple acids including ferulate, succinate, chlorogenic acid, and choline/O-phosphocholine. The negative association of FW with chlorogenic acid and anthocyanins were reported in our previous study^1,2^. Both phenotypic and genetic analysis revealed that fruit weight is a quantitatively inherited trait. Although we identified seven QTLs related to fruit size, none of them explained more than 10% of the PV and only three of the seven were detected more than one year, suggesting that the trait is governed by complex genotype by environmental factors^1,28^.

### Implications of fruit quality QTLs for blueberry breeding

Blueberry is a highly heterozygous plant with unique features including both asexual and sexual reproductive systems, production of gametes with unreduced chromosome number, a range of ploidy levels (diploid, tetraploid, and hexaploid), and a long juvenile period^15,17,19^. The history of blueberry breeding, and commercial cultivation goes back to the early 1900s. Given its recent domestication and breeding history, breeding programs have achieved much success including interspecific hybridization to develop cultivars adapted to warm climates and exploiting phenotypically diverse germplasm regarding specific traits^15,17^. However, the genetic mechanisms involved in major FQ and agronomic traits are by no means fully explored^11,28^.

This study provides the first insight into the inheritance of multiple fruit quality traits and detected major-effect loci for TA, TSS, developed a high-density linkage map with highest number of markers and individuals, and established the first assessment of the relationship between fruit quality traits and metabolites (sugars, organic acids, and amino acids). The traits evaluated here are complex and quantitative in nature. However, there are promising QTLs for marker-assisted selection for TA and TSS. The balance between TA and TSS is an important objective of blueberry breeding programs to address consumer preferences. The QTLs on chromosome 3 for TA and 7 and 10 for TSS could be a target for future fine mapping, functional gene analysis and then marker-assisted development and selection. In this study, we also highlighted the potential of the existing genetic variability to dissect the genetic basis of organic acids. Overall, the study provides critical information for blueberry breeding and future studies.

## Materials and methods

### Plant materials and phenotyping for FQ traits

The study included 287 F_1_ genotypes derived from a cross between ‘Reveille’ and ‘Arlen’. ‘Arlen’ is a paternal parent whereas ‘Reveille’ is a maternal parent. Both parental cultivars were unpatented and released by North Carolina State University^37^. The 287 F_1_ seedlings were grown in Ivanhoe, located within the state of North Carolina at coordinates of 34.5845° N, 78.2419° W (Sampson County), USA. Berries from the 287 F_1_ seedlings were harvested when fully ripe as indicated by the surface of the skin of the berries being completely blue for three consecutive years (2016–2018). After the harvest, the berries were stored at −80 °C until processing. Frozen berries (three replicates of approximately 10–30 g each), were then used to evaluate the following FQ traits: pH, TSS, TA, and fruit size. For the fruit harvested in 2016 and 2017, fruit size was estimated using an image-based method^1,38^. For the fruit harvested in 2018, image-based phenotyping and fruit weight (g per fruit) were highly correlated (*R*^2^ > 97%)^1^, average fruit weight was used as a proxy of fruit size.

For evaluating the other fruit quality traits (TSS, pH, and TA), the berries were homogenized to a puree in a Waring Commercial Blender 7012 G (Torrington, CT, United States). The homogenized samples were used to measure TSS, TA, and pH. TSS, TA, and pH were measured as described^1,2^. Briefly, TSS was measured using a digital hand-held ‘pocket’ refractometer PAL-1 (Atago, Tokyo, Japan) and the measurements were expressed as °Brix. The pH and TA were measured using 1 g of homogenized sample diluted with 30 ml pre-boiled double distilled water. The pH was measured using an Accumet AB15, pH-meter (Fisher Scientific, Waltham, MA, United States). Subsequently, the TA was determined with a Mettler DL15 Auto-Titrator (Columbus, OH, United States) at a pH of 8.2 using 0.02 mol L^–1^ sodium hydroxide and milliequivalent factor value 0.064. The TA was expressed as the percentage of citric acid (wt/wt) per 1 g FW.

### Extraction and quantification of metabolites

In addition to FQ traits, we randomly selected 98 F_1_ genotypes from the RxA mapping population and these were profiled for metabolites including sugars, amino acids, and organic acids. Berries from the 98 F_1_ genotypes were harvested when fully ripe during the 2016 cropping season. The berries were freeze-dried in 50 mL tubes and stored in −80 °C until further processing.

Approximately 1.4 g of freeze-dried berry sample was pulverized with a glass rod and brought to a total volume of 20 mL using 70% methanol, and then homogenized with a stick blender. The samples were centrifuged for 20 min at 20 °C at 5000 × *g*, and the supernatant filtered through glass wool into a 50 mL volumetric flask. Approximately 10 mL of 70% methanol was added to the pellet, vortexed and centrifuged and the supernatant combined in the volumetric flask with the previous fraction. The pellets were rinsed a second time as above, and the combined supernatant brought to a total volume of 50 mL with 70% methanol. A 1 mL aliquot of the extract was transferred to a tube and stored at −80 °C. After processing, all samples were thawed and evaporated under a stream of nitrogen to dryness. The dried extract was reconstituted in 650 µL of NMR solvent (100 mM phosphate buffer, 0.5 mM DSS, in 100% D2O, pH 7.4), and 600 μL was transferred to a 5 mm 7″ NMR tube for analysis. A 25 µL aliquot of each study sample was combined to generate a pooled QC sample, and 4 × 600 µL transferred into NMR tubes.

NMR spectra were recorded at 298 K on a Bruker 600 MHz Avance III spectrometer equipped with a DCH cryogenically cooled probe, operating at a 1H frequency of 600,133 MHz. For each of the study samples, data were collected using pulse sequences incorporated into the Bruker TOPSPIN 2.1 software. 1D 1H data were acquired (noesypr1d) with 192 scans across 32 K data points, sweep width 11 ppm, delay 2 s, noisy mixing time 100 msec. Free induction decays (FIDs) were Fourier transformed with 0.5 Hz line broadening and no zero-filling applied, and spectra were manually phased, and baseline corrected using only zero order correction, and NMR chemical shifts referenced to the DSS signal at 0.0 ppm. For the pooled sample, additional data were collected to assist with annotations (1D 13 C, 2D-HSQC, 2D-HMBC, COSY). Spectra for the pooled QC samples were used to segment 1H NMR spectra into domains and integrated using MestReNova NMR suite (version 11.0.3, Mestrelab Research S.L., Escondido, CA). All spectra were then integrated and normalized by area of the DSS peak and sample mass. Spectral peaks were annotated using standard reference libraries integrated with Chenomx NMR Suite software (version 13, Chenomx Inc., Edmonton, Canada) with further confirmation made when necessary by matching spectral data (1D/2D) to reference data (Biological Magnetic Resonance Data Bank (BMRB))^39^. Annotation confidence levels were derived from recommended minimum reporting standards for analytical data^40^. Briefly, the levels were divided as follows: ^1^H spectra matched to a reference library entry (2 A); Multiple peaks (1D and/or 2D) matched to reference library data (2B); Compound class assigned (3); Unknowns (4).

### Phenotypic data analysis

For QTL analysis in individual years, genotype means were calculated based on the average values of the three replicates. Genotype means over years were estimated using best linear unbiased estimate (BLUE), with both genotype and year considered as fixed factors. Broad-sense heritability was estimated using variance components calculated from the restricted maximum likelihood (REML), calculated as follows:

$$H^2 = \frac{{\delta g^2}}{{(\delta g^2 + \frac{{\delta gy^2}}{y} + \frac{{\delta e^2}}{{ry}})}}$$

where *δg*^2^, *δe*^2^, and *δgy*^2^ are variance components of genotypes, plot-to-plot variation of residuals and [genotype x environment] interaction, respectively; y is the number of environments (number of years in this study, =3) and r is the number of replications (=3). The relationship between traits was calculated using the Pearson Coefficient of Correlation using BLUE and three-year data, independently. The correlation was visualized using the R package corrplot^41^.

### DNA extraction, SNP detection, and SNP genotyping

Total genomic DNA from individual plants was extracted using 3 grams of fresh leaves by CTAB method^42^. DNA concentration and purity were evaluated using Qubit® and NanoDrop®. DNA sequencing was performed by RAPiD Genomics (Gainesville, FL, USA) using capture-seq method. Prior to the genotyping of the F_1_ clones, the parents (‘Reveille’ and ‘Arlen’) were evaluated using a set of 31,000 probes. Subsequently, 10k informative probes with the maximum number of variants evenly distributed across the genome were selected for genotyping the F_1_ clones. The capture-seq was used to make Illumina paired-end libraries. An equimolar of the population’s individual libraries were pooled and ran on two lanes of Illumina 2500.

Raw reads of all individuals’ pooled libraries were demultiplexed. The low-quality reads were removed, and the remaining reads were trimmed for adapters and primer sequences using Trimmomatic^43^. High-quality short reads from each library were mapped against the blueberry reference genome sequence using BWA MEM aligner^44^. Uniquely mapped reads were used to call SNPs using freebayes v.1.0.1^45^, targeting 1,000 bp flanking the 10k probe regions. Following the SNP calling, the dataset was filtered: (I) minimum mapping quality of 20; (II) mean depth of coverage of 50; (III) maximum missing data of 10% across SNPs and individuals; (IV) only biallelic loci. Then, the read depth of the reference and alternative alleles of each SNP and individuals was extracted from the variant call file using vcftools v.0.1.16^46^. The tetraploid allele dosages were called based on the read depth counts and “F_1_” model using updog R package^47^. The genotypes for the tetraploid calling were coded as 0 for nulliplex (AAAA), 1 for simplex (AAAB), 2 for duplex (AABB), 3 for triplex (ABBB), and 4 for quadruplex (BBBB).

### Linkage map construction

The linkage map was constructed using an R package PolymapR^24^. Prior to linkage map construction, a chi-square test for goodness of fit was performed at *P* < *0.01* and *P* < *0.05* for simplex (simplex by nulliplex) and higher dosage SNPs (duplex by nulliplex, duplex by simplex, duplex by duplex), respectively. Furthermore, the quality of markers and individuals were assessed based on the individual/SNP missing value rate. Individuals/markers with more than 10% missing value rate were discarded prior to linkage analysis. The linkage map was constructed per parent. For each parent simplex markers were used to estimate the two-point recombination frequency between markers. Following pairwise linkage analysis, the markers were clustered into 48 homologs using LOD scores. Then, the 48 homologs (four homologs per linkage group are expected in a tetraploid) were assigned to the 12 chromosomes using duplex by nulliplex markers. Duplex by nulliplex (D×N) markers were used to provide bridging linkages between homologous clusters. Following this step, double simplex (S×S) and higher dosage markers including double by simplex (D×S), duplex by duplex (D×D), and simplex by duplex (S×D) were added to the linkage groups based on the two-point linkage analysis. Finally, the markers were ordered using multidimensional scaling (MDS) and visual inspection of outliers^48^. Markers with nearest neighbor fit >5 were discarded as recommended^48^. Up to five rounds of MDSmap were performed until all outlying markers were removed. Finally, all linkage groups were combined to develop an integrated linkage map. Graphical presentations of linkage maps were prepared using Mapchart 2.3^49^ and ggplot2 R package^50^.

The resulting linkage map was aligned to the reference Draper blueberry genome^25^. The order of markers on the genetic map was reversed if the orientation of the physical and genetic map was found to be inversely ordered in respect to the reference genome. Meiotic recombination rate was estimated using loess (locally weighted regression) smoothing with a span of 0.4 using MareyMap^51^. This regression smooths over windows of a fixed number of SNPs instead of physical length to reduce bias in regions where more SNPs were recovered.

### QTL mapping for FQ traits

QTL analysis was performed using TetraploidSNPMap^23^. The phenotypic data were regressed to the QTL genotypes at each position, with the regression coefficients being weighted by the conditional probabilities of the QTL genotypes. The trait value was modeled as an additive function of the QTL allele effect on each of the eight homologous chromosomes (referred to in the results as the “full model”) as described^52^. The full model is an additive function of the QTL allele effect on each of the eight homologous chromosomes for each position on the grid. This model was fitted by regression of the trait values on the QTL genotype probabilities from the Hidden Markov Model (HMM). For each trait, a significant QTL was declared with 1,000 permutations and 95% confidence interval thresholds. For each significant QTL, the “simpler model” function was tested, and the best simple model was identified using the Schwarz Information Criteria (SIC)^53^. The SIC is calculated in TPM as follows:

$${\mathrm{SIC}} = - 2{\mathrm{logL}} + {\mathrm{plogmo,}}$$

where L is the likelihood of the simple model, p is the number of parameters in the simple model and mo is the number of observations (the 36 genotype means). The best simple model was represented by the lowest value for the SIC and the best simple model was considered a good fit if the SIC was lower than that of the full model, and the adjusted R^2^ values of the simple model were close to or better than the full model^52^. For presentation, homologous chromosomes derived from ‘Arlen’ are designated h1 to h4 and those derived from ‘Reveille’ are designated h5 to h8.

### Candidate gene annotation

Where possible, we reported the potential candidate genes for the QTLs that had been detected at least for two years and explained more than 10% of the PV. In order to define the regions of the genome, we first identified the best SNP configuration from simple model analysis. Then, the sequence of the SNP was blasted against the Draper genome (*V. corymbosum* cv. Draper v1.0 genome scaffolds). We searched for candidate genes within 100 kb regions of the genome on both the right and left side of the SNP. The function of the genes that were found in the 100 kb regions were blasted against the NCBI.

## Supplementary information

Supplementary Figure 1-8 - revised

Supplementary Table S1-S5 - revised

## References

[1] Mengist MF (2020). Diversity in metabolites and fruit quality traits in blueberry enables ploidy and species differentiation and establishes a strategy for future genetic studies. Front. Plant Sci..

[2] Mengist MF (2020). Development of a genetic framework to improve the efficiency of bioactive delivery from blueberry. Sci. Rep..

[3] Farneti B (2020). Development of a novel phenotypic roadmap to improve blueberry quality and storability. Front. Plant Sci..

[4] Gilbert JL (2015). Identifying breeding priorities for blueberry flavor using biochemical, sensory, and genotype by environment analyses. PLoS ONE.

[5] Evans E. A. & Ballen F. H. An overview of US blueberry production, trade, and consumption, with special reference to Florida. *University of Florida, IFAS, Extension, FE952**Available online*: https://edis.ifas.ufl.edu/publication/fe952 2014.

[6] Yang B, Kortesniemi M (2015). Clinical evidence on potential health benefits of berries. Curr. Opin. Food Sci..

[7] Gallardo RK (2018). Breeding trait priorities of the blueberry industry in the United States and Canada. HortScience.

[8] Brazelton C., Kayla Y. & Bauer N. 2016 Global Blueberry Statistics and Intelligence Report. 2017. https://www.internationalblueberry.org.

[9] Rawandoozi ZJ (2020). Identification and characterization of QTLs for fruit quality traits in peach through a multi-family approach. BMC genomics.

[10] Calle A, Wünsch A (2020). Multiple-population QTL mapping of maturity and fruit-quality traits reveals LG4 region as a breeding target in sweet cherry (Prunus avium L.). Horticulture Res..

[11] Benevenuto J, Ferrão LFV, Amadeu RR, Munoz P (2019). How can a high-quality genome assembly help plant breeders?. Gigascience.

[12] Zeng Q (2020). High altitude is beneficial for antioxidant components and sweetness accumulation of Rabbiteye Blueberry. Front. Plant Sci..

[13] Zhang J (2020). Evaluation of sugar and organic acid composition and their levels in highbush blueberries from two regions of China. J. Integr. Agriculture.

[14] Colantonio V (2020). Metabolomic selection for enhanced fruit flavor. bioRxiv.

[15] Rowland LJ (2012). Generating genomic tools for blueberry improvement. Int. J. Fruit. Sci..

[16] Lobos GA, Hancock JF (2015). Breeding blueberries for a changing global environment: a review. Front. Plant Sci..

[17] Retamales JB, Hancock JF (2018). Blueberries..

[18] Rowland LJ, Levi A (1994). RAPD-based genetic linkage map of blueberry derived from a cross between diploid species (Vaccinium darrowi and V. elliottii). Theor. Appl. Genet.: Int. J. Plant Breed. Res..

[19] Hancock JF, Lyrene P, Finn CE, Vorsa N, Lobos GA (2008). Blueberries and cranberries. Temp. Fruit. Crop Breed.: Germplasm Genomics.

[20] Rowland LJ, Ogden EL, Vinyard BT (2020). Phenotypic evaluation of a hybrid diploid blueberry population for plant development and fruit quality traits. Agronomy.

[21] Cappai F (2020). High-resolution linkage map and QTL analyses of fruit firmness in autotetraploid blueberry. Front. Plant Sci..

[22] Mengist MF (2018). Genetic mapping of quantitative trait loci for tuber-cadmium and zinc concentration in potato reveals associations with maturity and both overlapping and independent components of genetic control. Theor. Appl. Genet..

[23] Hackett CA, Boskamp B, Vogogias A, Preedy KF, Milne I (2017). TetraploidSNPMap: Software for linkage analysis and QTL mapping in autotetraploid populations using SNP dosage data. J. Heredity.

[24] Bourke PM (2018). polymapR—linkage analysis and genetic map construction from F1 populations of outcrossing polyploids. Bioinformatics.

[25] Colle M (2019). Haplotype-phased genome and evolution of phytonutrient pathways of tetraploid blueberry. GigaScience.

[26] McCallum S (2016). Construction of a SNP and SSR linkage map in autotetraploid blueberry using genotyping by sequencing. Mol. Breed..

[27] Zhou Q (2020). Haplotype-resolved genome analyses of a heterozygous diploid potato. Nat. Genet..

[28] Ferrão LFV (2018). Insights into the genetic basis of blueberry fruit-related traits using diploid and polyploid models in a GWAS context. Front. Ecol. Evolution.

[29] Wu B (2017). Genome-wide identification, expression patterns, and functional analysis of UDP glycosyltransferase family in peach (Prunus persica L. Batsch). Front. Plant Sci..

[30] Hong Z, Zhang Z, Olson JM, Verma DPS (2001). A novel UDP-glucose transferase is part of the callose synthase complex and interacts with phragmoplastin at the forming cell plate. Plant Cell.

[31] Moremen KW, Haltiwanger RS (2019). Emerging structural insights into glycosyltransferase-mediated synthesis of glycans. Nat. Chem. Biol..

[32] Shi C-Y (2015). Citrus PH5-like H+-ATPase genes: identification and transcript analysis to investigate their possible relationship with citrate accumulation in fruits. Front. Plant Sci..

[33] Cohen S (2014). The PH gene determines fruit acidity and contributes to the evolution of sweet melons. Nat. Commun..

[34] Verma, S. et al. Two large-effect QTLs, Ma and Ma3, determine genetic potential for acidity in apple fruit: breeding insights from a multi-family study. *Tree Genetics and Genomes* 2019; **15**10.1007/s11295-019-1324-y.

[35] Fong, S. K. et al. A low malic acid trait in cranberry fruit: genetics, molecular mapping, and interaction with a citric acid locus. *Tree Genetics and Genomes* 2021;**17**10.1007/s11295-020-01482-8.

[36] Diaz-Garcia L (2018). Massive phenotyping of multiple cranberry populations reveals novel QTLs for fruit anthocyanin content and other important chemical traits. Mol. Genet. Genomics.

[37] Cline, B. 2007 Blueberry Workshop Agent Training Sponsored by: NC Blueberry Council. 2007 https://smallfruits.org/files/2019/06/SRFC-training-Jun-19-21-2007.pdf.

[38] Diaz-Garcia L (2016). GiNA, an Efficient and high-throughput software for horticultural phenotyping. PLoS ONE.

[39] Ulrich EL (2007). BioMagResBank. Nucleic acids Res..

[40] Sumner LW (2007). Proposed minimum reporting standards for chemical analysis. Metabolomics.

[41] Wei T (2017). Package ‘corrplot’. Statistician.

[42] Panta GR, Rowland LJ, Arora R, Ogden EL, Lim CC (2004). Inheritance of cold hardiness and dehydrin genes in diploid mapping populations of blueberry. J. Crop Improv..

[43] Bolger A (2014). The genome of the stress-tolerant wild tomato species. Nat. Genet..

[44] Li H, Durbin R (2009). Fast and accurate short read alignment with Burrows–Wheeler transform. bioinformatics.

[45] Garrison E., Marth G. Haplotype-based variant detection from short-read sequencing. *arXiv preprint arXiv:12073907* 2012.

[46] Danecek P (2011). The variant call format and VCFtools. Bioinformatics.

[47] Gerard D, Ferrão LFV, Garcia AAF, Stephens M (2018). Genotyping polyploids from messy sequencing data. Genetics.

[48] Preedy KF, Hackett CA (2016). A rapid marker ordering approach for high-density genetic linkage maps in experimental autotetraploid populations using multidimensional scaling. Theor. Appl. Genet..

[49] Voorrips RE (2002). MapChart: software for the graphical presentation of linkage maps and QTLs. J. Heredity.

[50] Wickham H. *ggplot2: elegant graphics for data analysis*. springer, 2016.

[51] Rezvoy C, Charif D, Guéguen L, Marais GAB (2007). MareyMap: an R-based tool with graphical interface for estimating recombination rates. Bioinformatics.

[52] Hackett CA, Bradshaw JE, Bryan GJ (2014). QTL mapping in autotetraploids using SNP dosage information. Theor. Appl. Genet..

[53] Schwarz G (1978). Estimating the dimension of a model. Ann. Stat..

