# Supplementary material for: High-density linkage map construction and identification of loci regulating fruit quality traits in blueberry
Source: Hortic Res. 2021 Aug 1;8:169. doi: 10.1038/s41438-021-00605-z (PMC8325695; doi:10.1038/s41438-021-00605-z)
Supplement: Supplementary file 1 — Supplementary Figure 1-8 - revised [file 41438_2021_605_MOESM1_ESM.docx]

**High density linkage map construction and identification of loci regulating fruit quality traits in blueberry.**

Molla F. Mengist^1^, Hamed Bostan^1^, Elisheba Young^2^, Kristine Kay^3^, Hamid Ashrafi^2^, Nicholas Gillitt^3^, James Ballington^2^, Mario G. Ferruzzi^1,4^, Mary Ann Lila^1,4^, Massimo Iorizzo^1, 2*^

^1^ Plants for Human Health Institute, North Carolina State University, 600 Laureate Way, Kannapolis, NC, 28081, USA

^2^ Department of Horticultural Science, North Carolina State University, Raleigh, North Carolina, USA

^3^ David H. Murdock Research Institute, 150 N Research Campus Dr, Kannapolis, NC 28081

^4^ Department of Food Bioprocessing and Nutrition Sciences, North Carolina State University, Raleigh, North Carolina, USA

*Correspondence: [miorizz@ncsu.edu](mailto:miorizz@ncsu.edu)


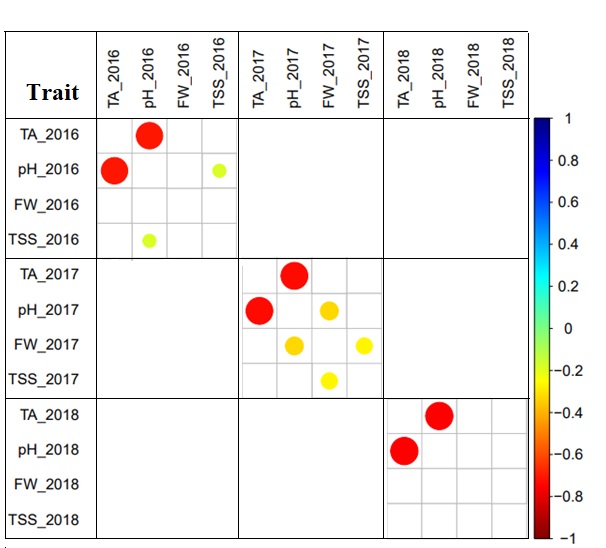


**Supplementary Figure 1**. Correlation between fruit quality traits over three years. _2016, _2017, _2018 refer the harvest years 2016,2017 and 2018, respectively. TA,titratable acidity; TSS, total soluble solid; FW, fruit weight .


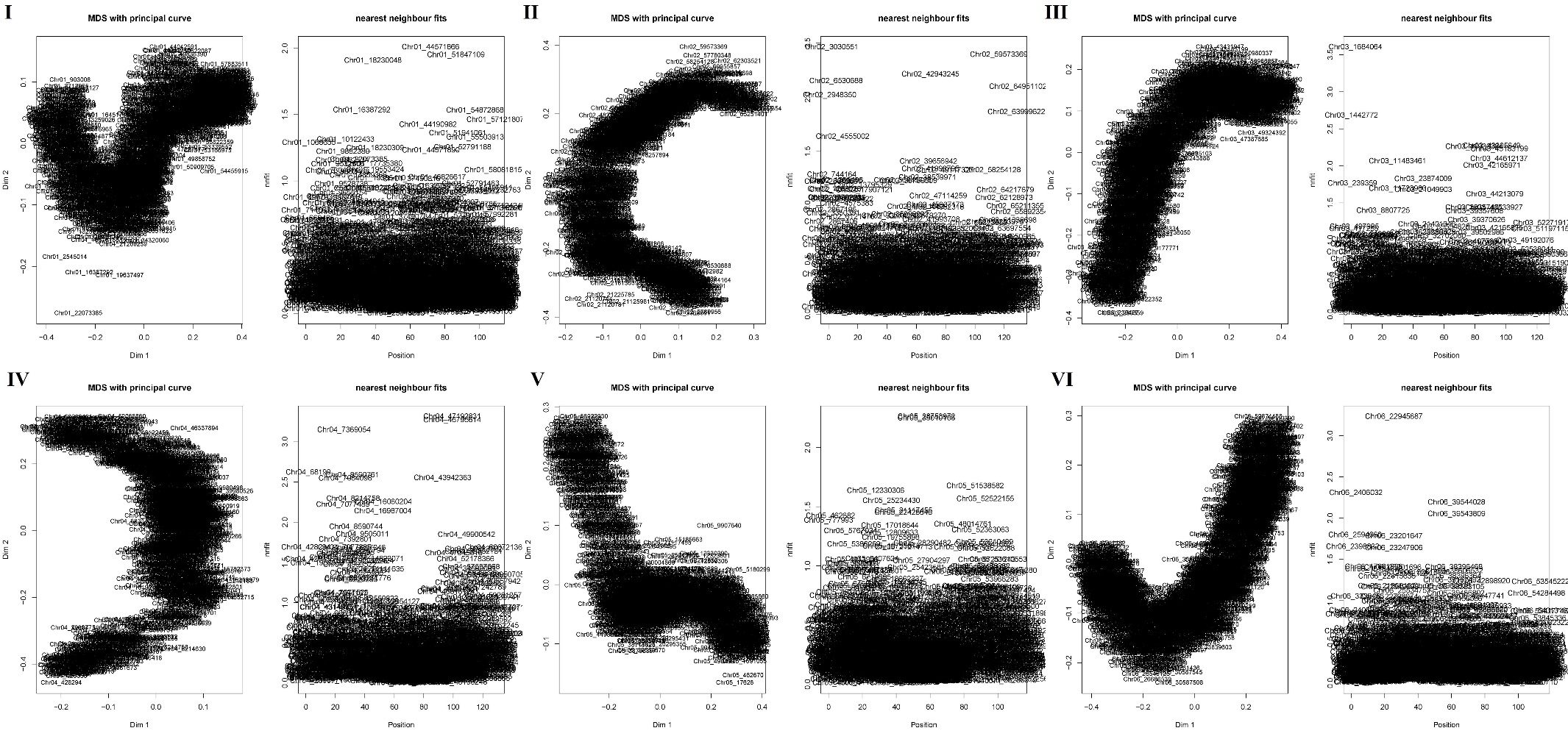


**Supplementary Figure 2**. Multidimensional scaling diagnostic plot representing configurations for SNP markers mapped on Reveille x Arlen chromosomes I to VI from the principal curves map estimation and nearest neighbor fits.


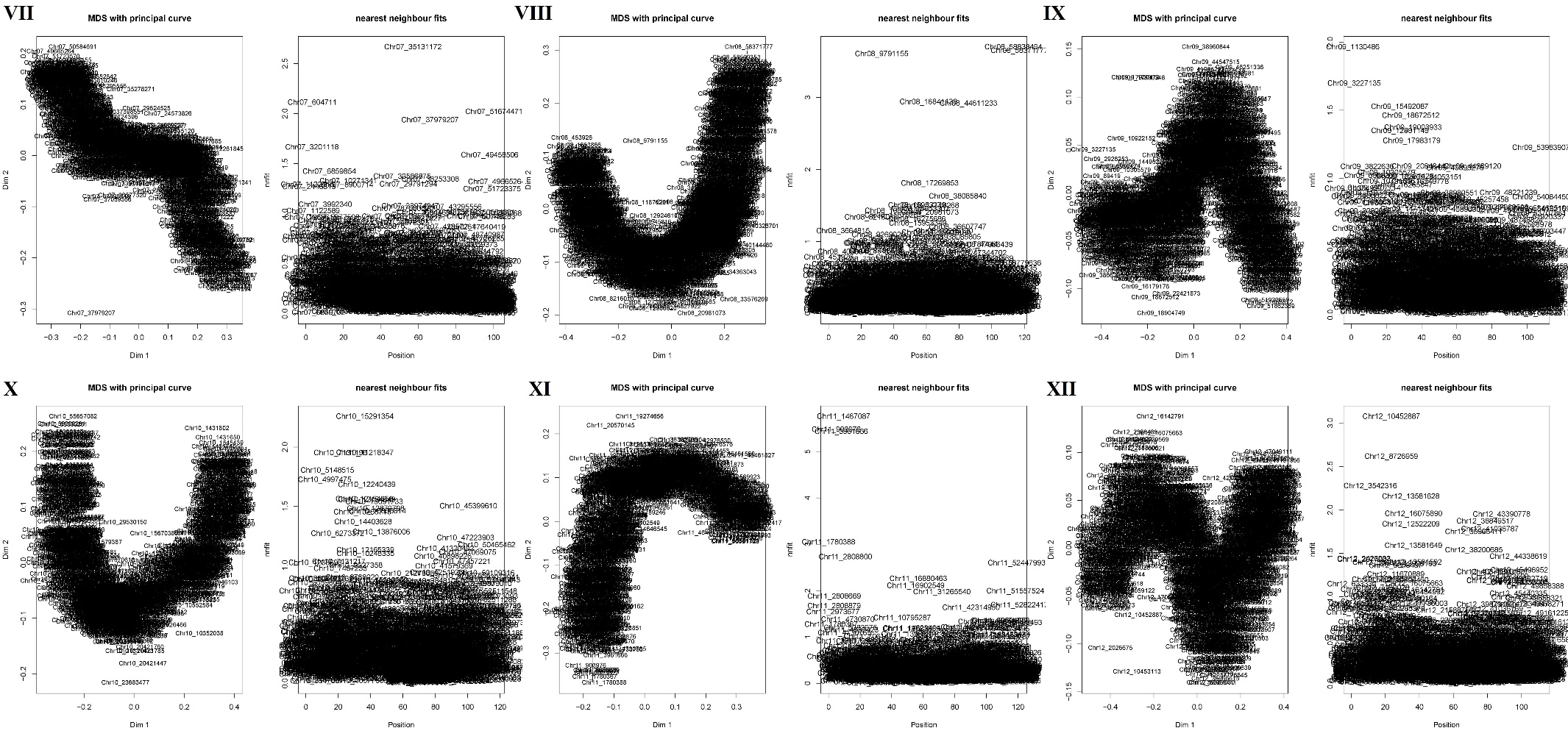


**Supplementary Figure 2, continue**. Multidimensional scaling diagnostic plot representing configurations for SNP markers mapped on Reveille x Arlen chromosomes VII to XII from the principal curves map estimation and nearest neighbor fits.


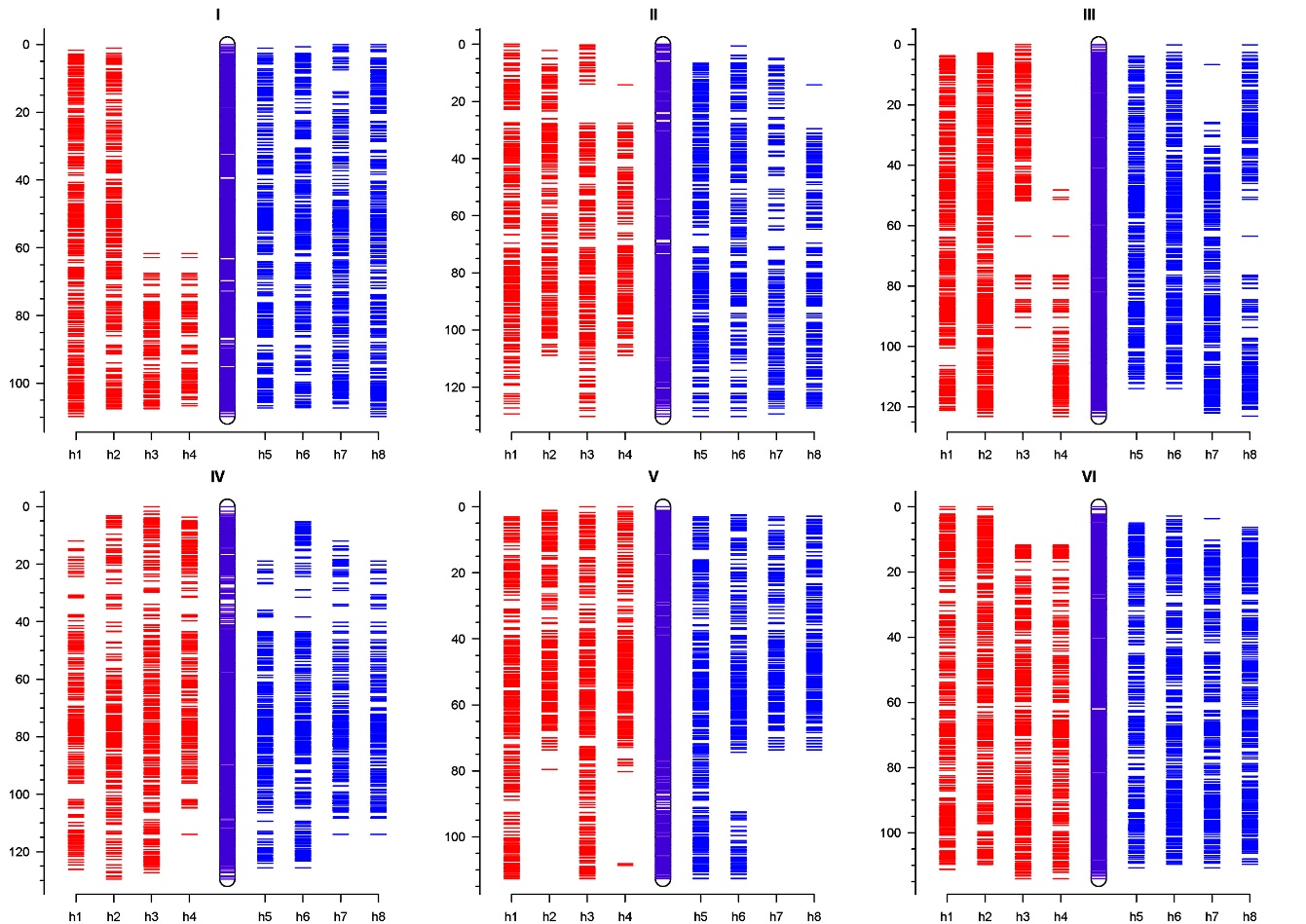


**Supplementary Figure 3**. Distribution of SNP markers across eight homologues of chromosomes I-VI. The four homologues represented in red (h1-h4) represent the parent Arlen (h1-4), the four homologues in blue (h5-h8) represent the parent Reveille.


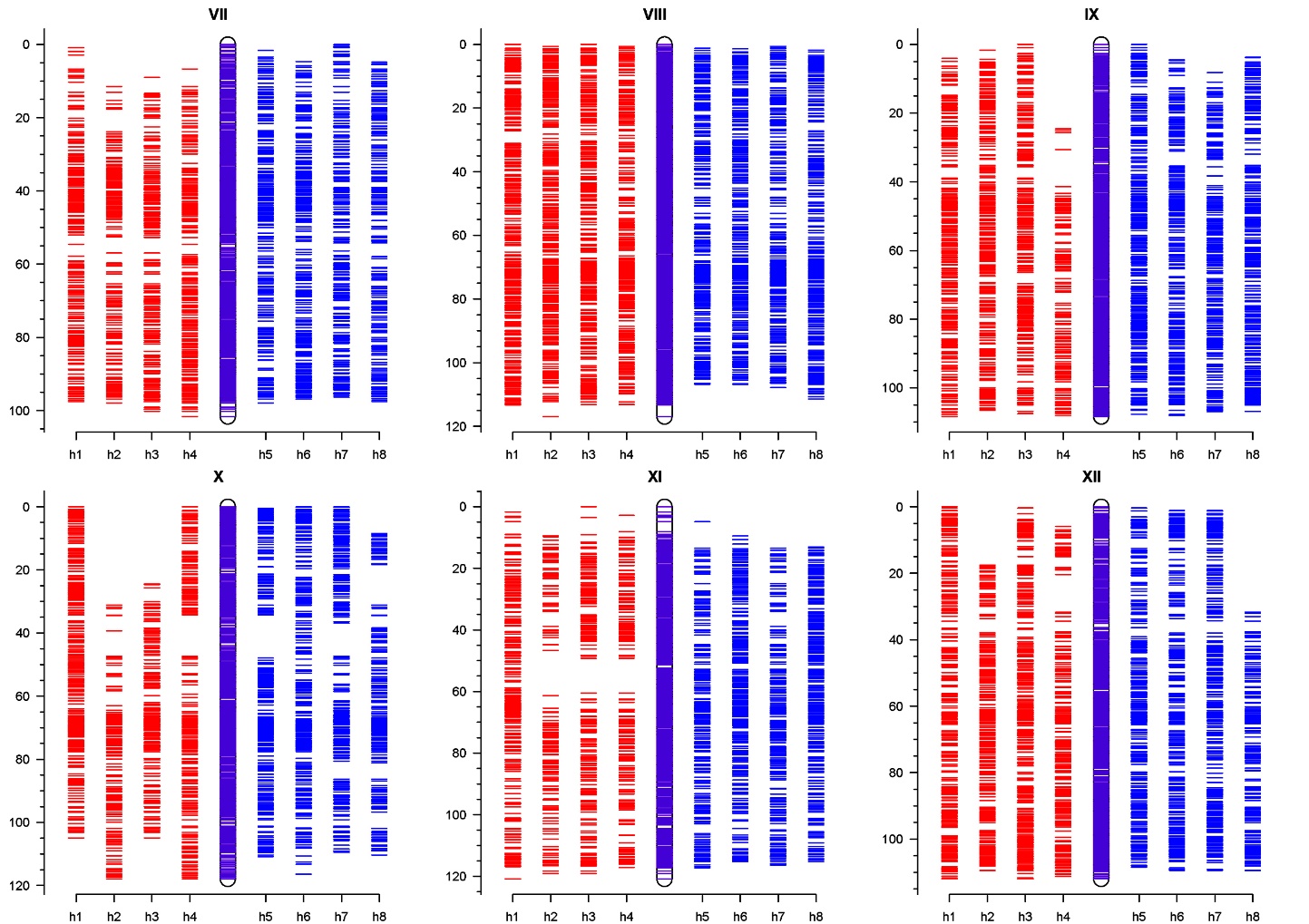


**Supplementary Figure 3, continue**. Distribution of SNP markers across eight homologues of chromosomes VII-XII. The four homologues represented in red (h1-h4) represent the parent Arlen (h1-4), the four homologous in blue (h5-h8) represent the parent Reveille.


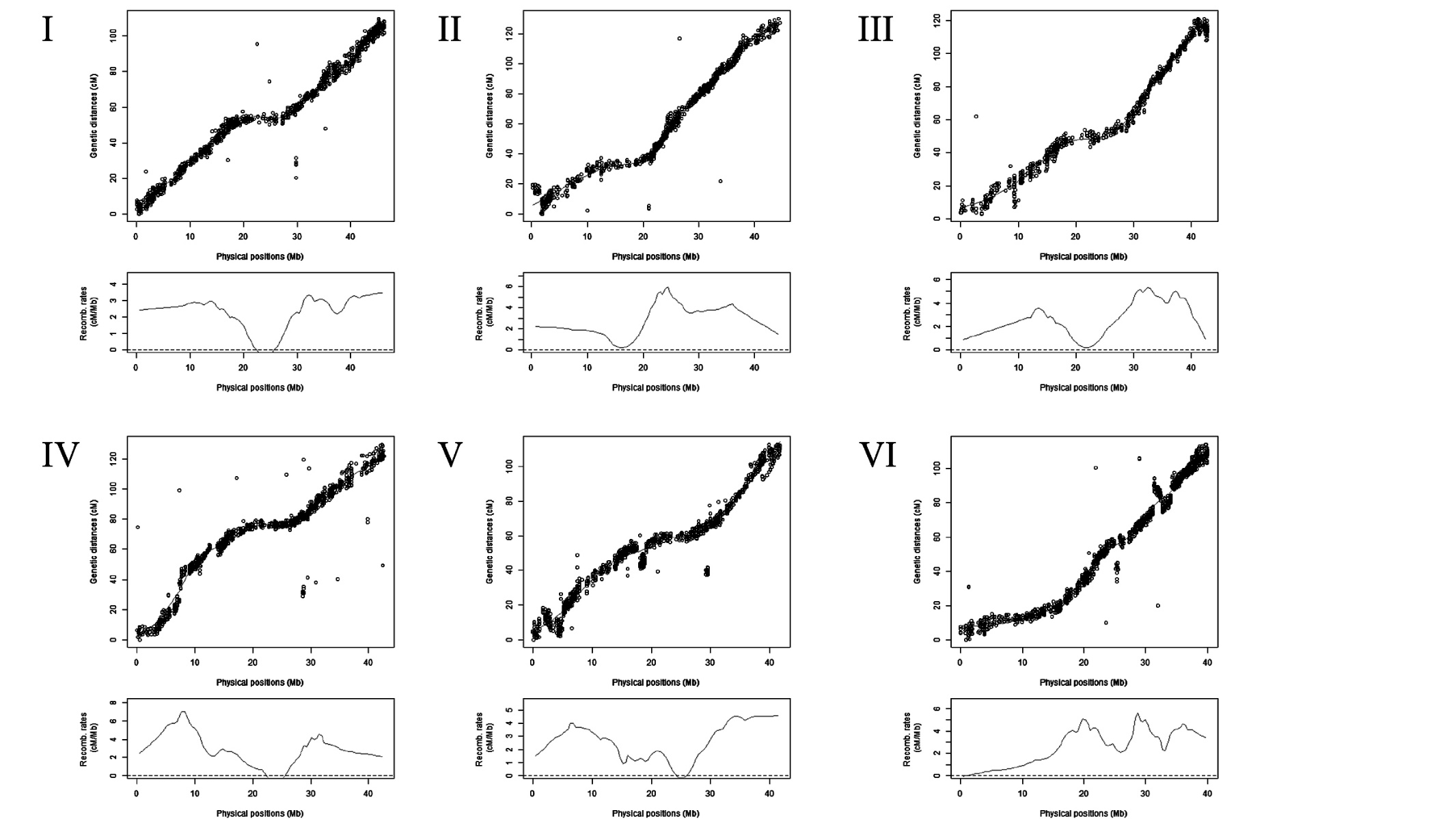


**Supplementary Figure 4**. Collinearity between the Reveille x Arlen genetic map (cM) and the physical map (Mb) of the Draper genome (Colle et al., 2019).


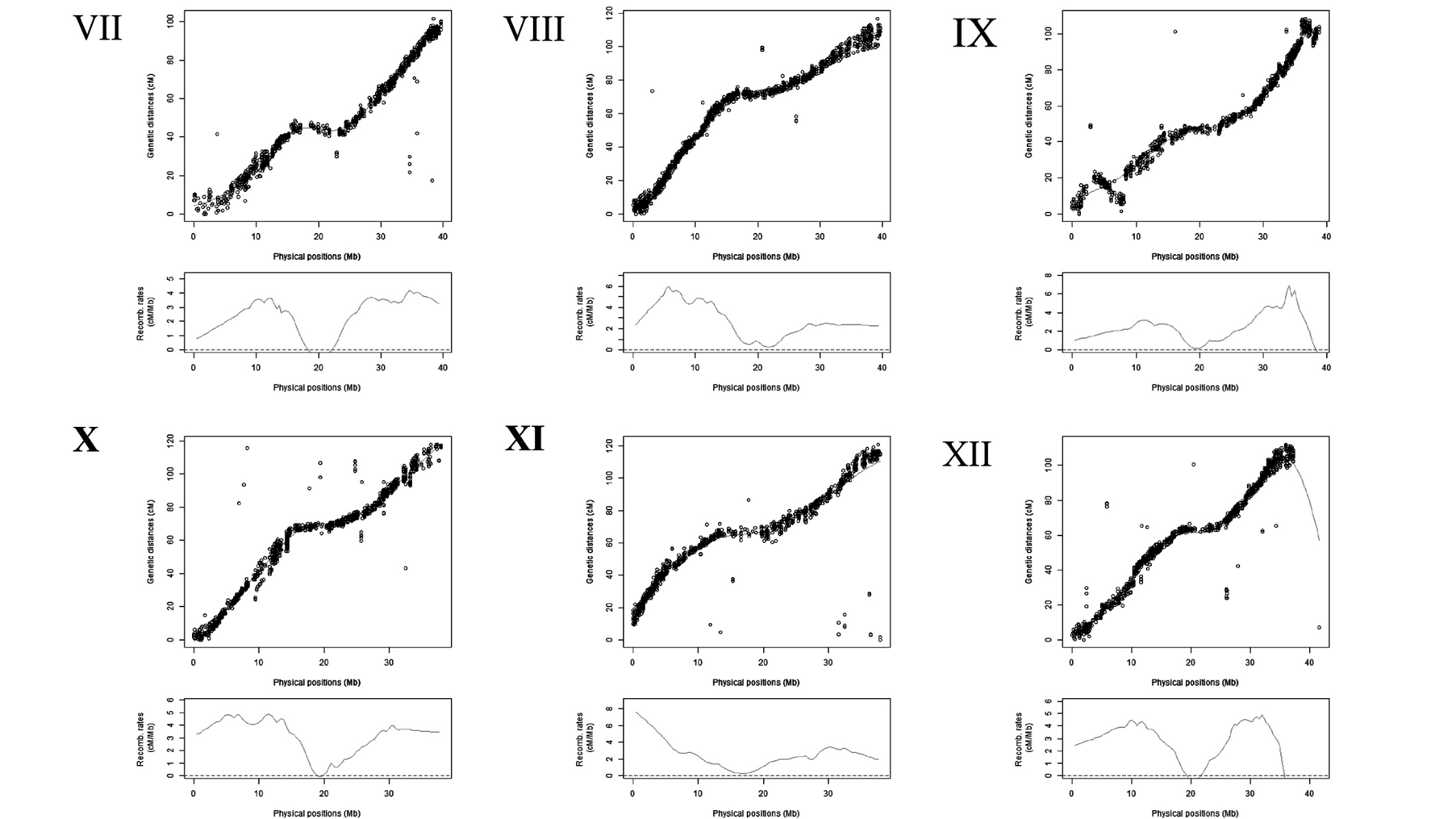


**Supplementary Figure 4, continue**. Collinearity between the Reveille x Arlen genetic map (cM) and physical map (Mb) of the Draper genome (Colle et al., 2019).


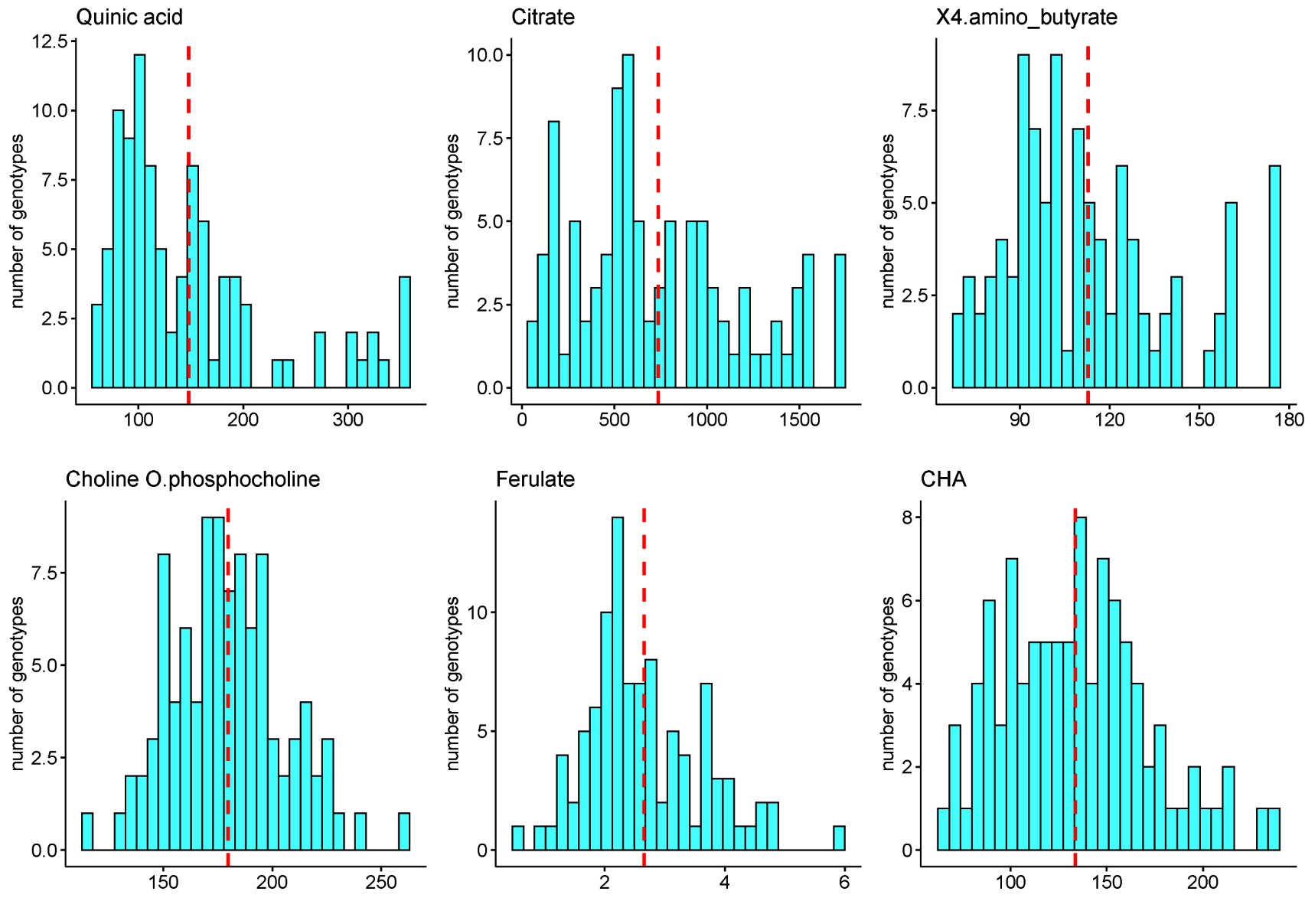


**Supplementary Figure 5**. Phenotypic distribution of metabolites detected by Proton nuclear magnetic resonance (^1^H NMR).


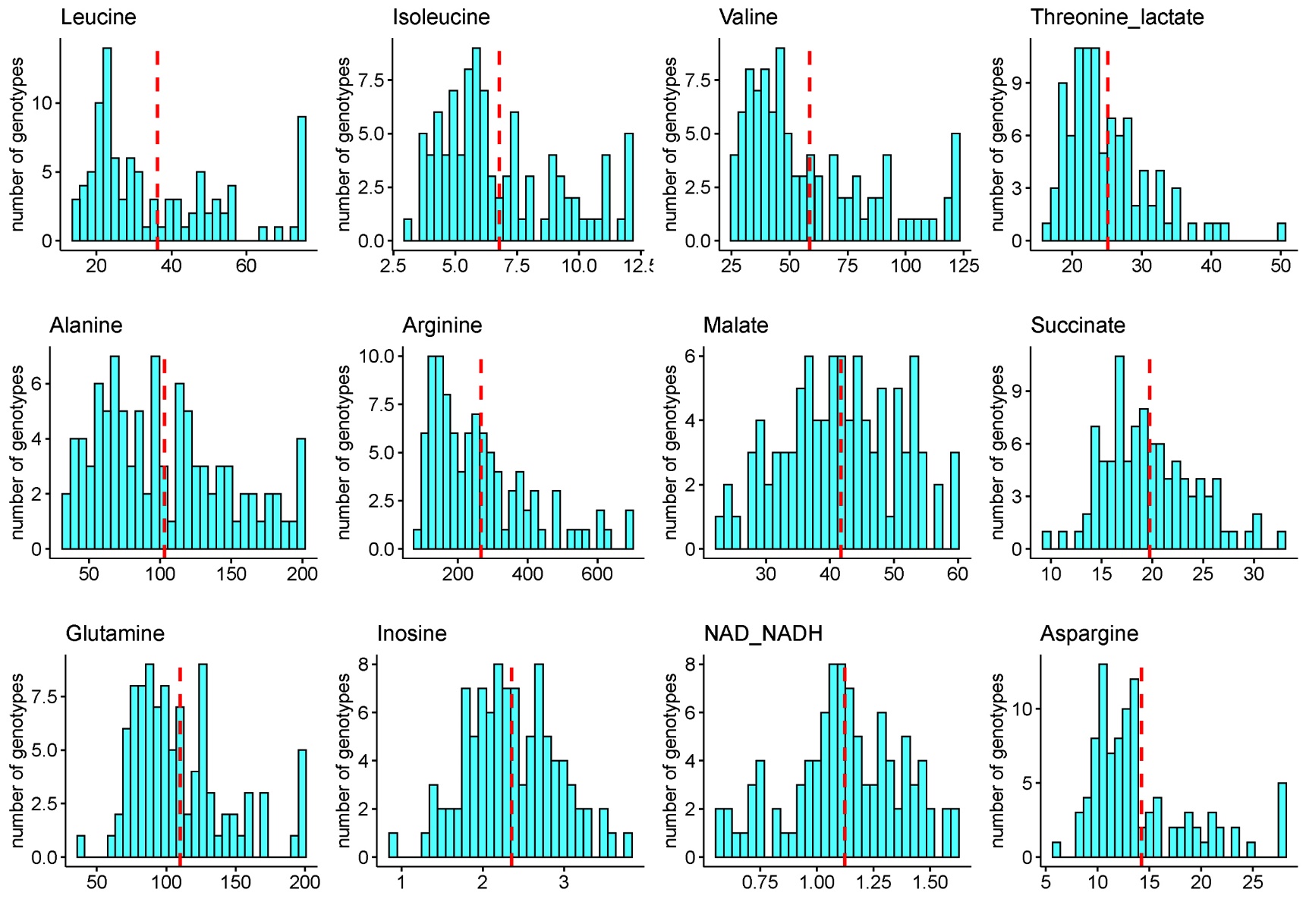


**Supplementary Figure 5, continue**. Phenotypic distribution of metabolites detected by Proton nuclear magnetic resonance (^1^H NMR).


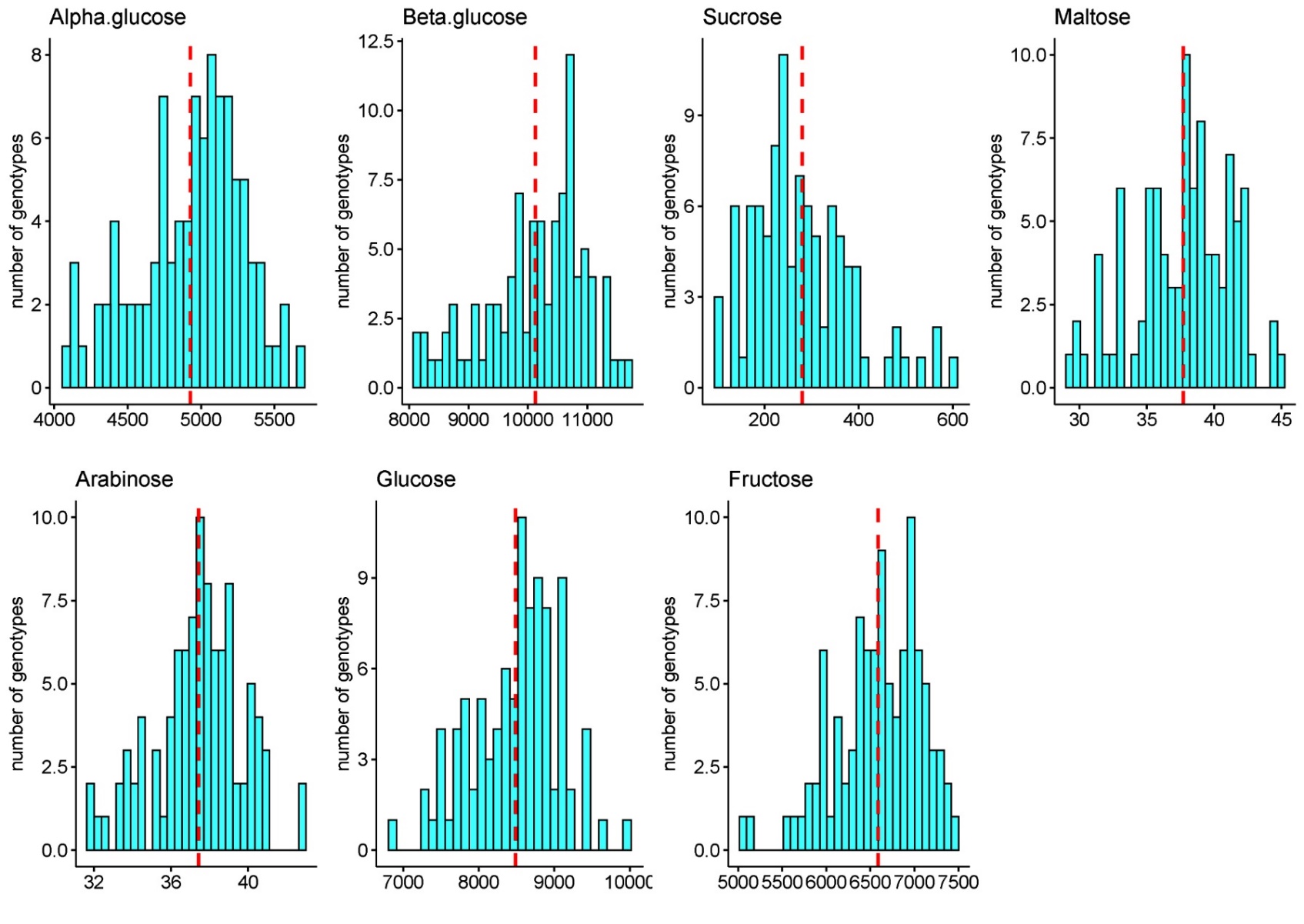


**Supplementary Figure 5, continue**. Phenotypic distribution of metabolites detected by Proton nuclear magnetic resonance (^1^H NMR).


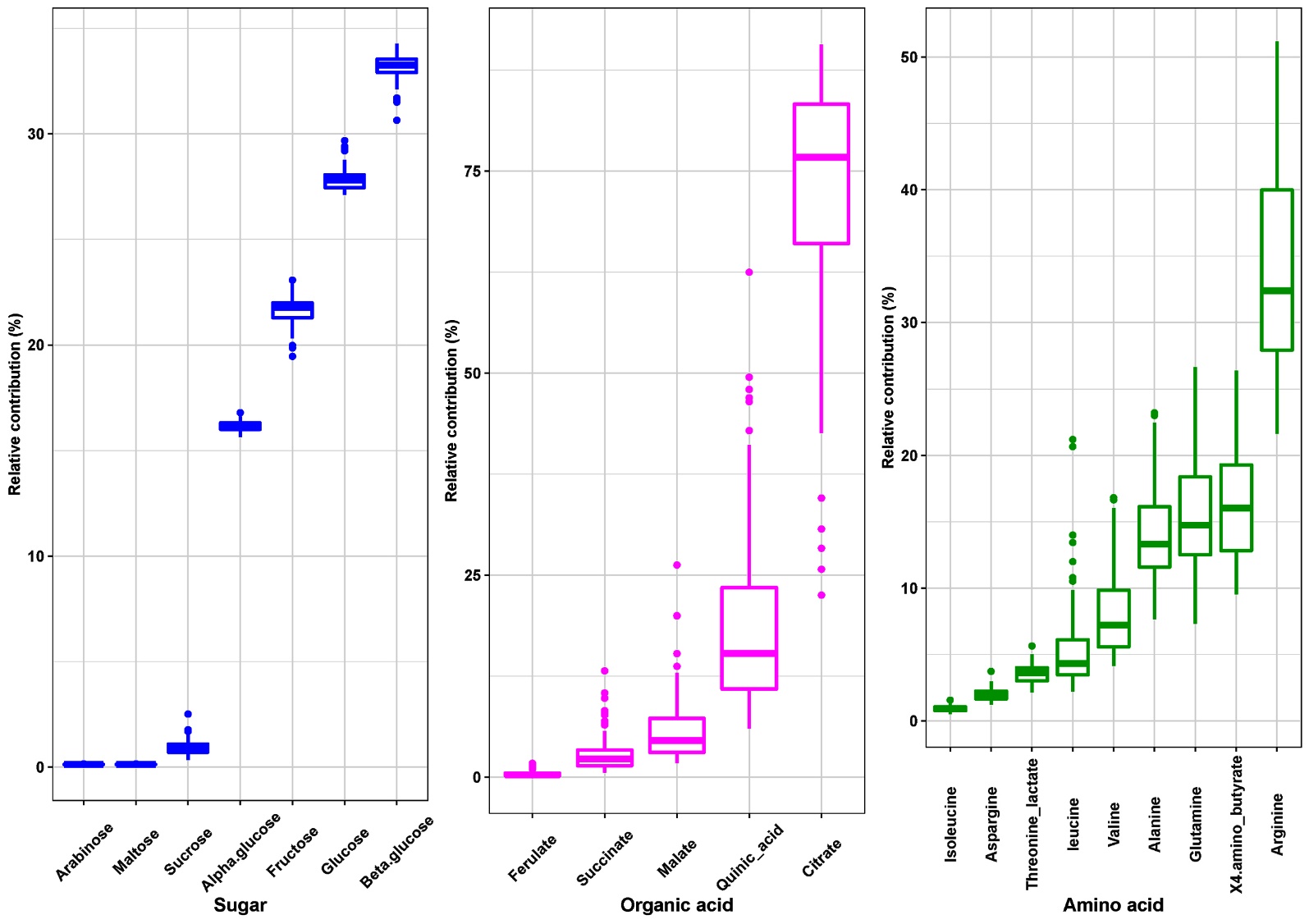


**Supplementary Figure 6**. Average relative composition (%) of sugars, organic acids, and amino acids detected across 98 F_1_ Reveille x Arlen genotypes.


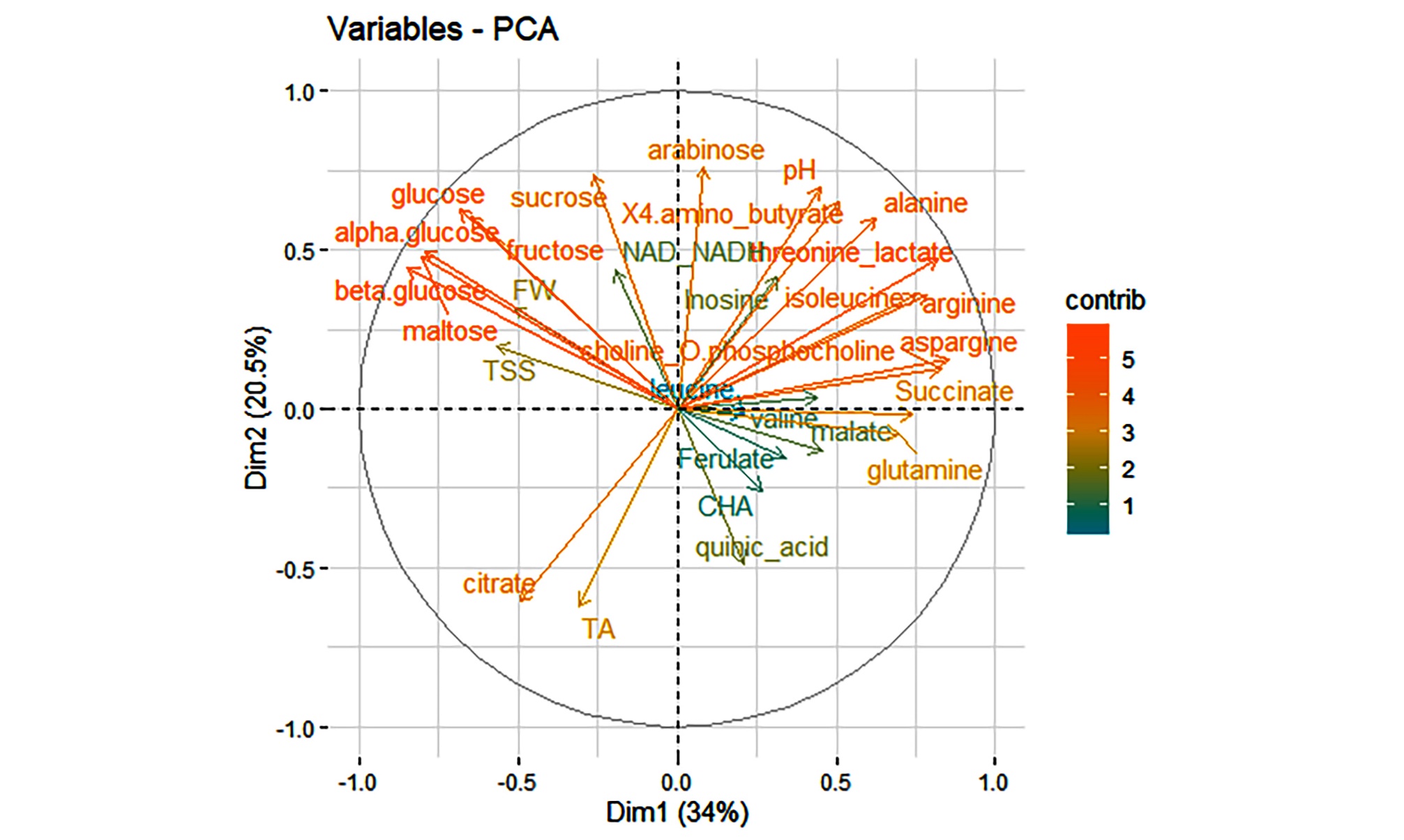


**Supplementary Figure 7**. Principal component analysis (PCA) of metabolites and fruit quality traits using data from 98 Reveille x Arlen F_1_ genotypes.


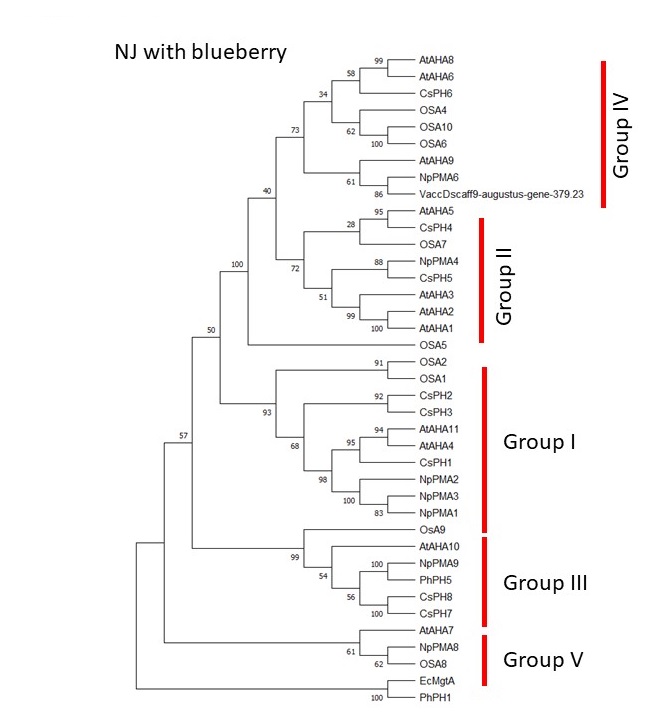


**Supplementary Figure 8**. Phylogenetic analysis of P-ATPases from blueberry. The phylogenetic tree was constructed using the MEGA 4.0 program with the neighbor-joining method. The numbers at the branch points indicate bootstrap support (1000 replicates).
